# For-profit outsourcing and its effects on placement stability and locality for children in care in England, 2011–2022: A longitudinal ecological analysis

**DOI:** 10.1016/j.chiabu.2023.106245

**Published:** 2023-10

**Authors:** Anders Malthe Bach-Mortensen, Benjamin Goodair, Jane Barlow

**Affiliations:** a Department of Social Policy and Intervention, University of Oxford, Barnett House, 32-37 Wellington Square, Oxford OX1 2ER, United Kingdom; b Department of Social Sciences and Business, Roskilde University, Roskilde, Denmark

**Keywords:** Children's social care, Placements, Quality, Outsourcing, Commissioning, Profit

## Abstract

**Background:**

The responsibility of local authorities in England to provide children in care with stable, local placements has become increasingly difficult due to the rising number of children in need of care and a shortage of available placements. It is unclear if the trend of outsourcing children's social care to private companies has exacerbated this challenge. This paper examines how the outsourcing of children's social care to the private market has influenced placement locality and long-term stability over time.

**Methods:**

We created a novel dataset of multiple administrative data sources on the outsourcing, placement locality and stability, and characteristics of children in care between 2011 and 2022. We conducted time-series fixed-effects regression analysis of the impact of for-profit outsourcing on placement locality and stability from 2011 to 2022.

**Results:**

Our fully adjusted models demonstrate that for-profit outsourcing is consistently associated with more children being placed outside their home local authority and greater placement instability. We found that an increase of 1 % point of for-profit outsourcing was associated with an average increase of 0.10 % points (95 % CI 0.02–0.17; *p* = 0.01) more children experiencing placement disruption, and 0.23 % points (95 % CI 0.15–0.30; *p* < 0.001) more children being placed outside their home local authority. We estimate that an additional 17,001 (95 % CI 9015–24,987) out-of-area placements can be attributed to increases in for-profit provision.

**Discussion:**

Our analyses show that placement stability and distance have deteriorated or stagnated over the last decade, and that the local authorities that rely most on outsourcing have the highest rates of placement disruptions and out-of-area placements.

## Highlights

• Social care services struggle to find children suitably located and stable care.
• England's services are frequently, and increasingly, outsourced to for-profits.
• For-profit outsourcing is linked with more placements away from the child's home.
• For-profit outsourcing is linked with more unstable, short-term placements.
• Further outsourcing to for-profits may not improve quality of care for children.

## 1 Introduction

The children's social care sector in England is struggling to meet the needs of children in care. In the last decade, several large-scale investigations have been commissioned by the UK government, including the 2016 Narey independent review of residential care (Narey, 2016), the children's social care market report by the Competition and Markets Authority (CMA) (CMA, 2022), and most recently, the independent review of children's social care (MacAlister, 2022). These reports all identify persistent flaws in the provision of children's social care.

Possibly the most important challenges for the sector is meeting the needs of increasing numbers of children in care, which is currently (2022) at a record high of 82,170 (Department for Education, 2022). The persistent growth in children in care has put great pressure on local authorities (LAs), who struggle to secure and commission accommodation for children in need (Bach-Mortensen, Murray, et al., 2022; CMA, 2022). The challenges associated with meeting this increasing demand are caused by a multitude of factors, but in particular by an insufficient supply and access to appropriate placements, especially for children with complex needs (CMA, 2022; Ofsted, 2021b). Ofsted recently declared that the sector is suffering a “sufficiency crisis” (Ofsted, 2021b) and that the demand for children's social care places “[…] consistently outstrips supply” (Ofsted, 2021a).

The severe shortage of appropriate and available children's social care placements is detrimental to the placement process for children in care. As per the statutory guidance, placement decisions should be based on the appropriateness of the match between the expertise of a provider and the needs of a child (Department for Education, 2010). Further, it is the responsibility of local authorities to provide accommodation within their area that meets the needs of the child and allows them to live near their home (Department for Education, 2022). However, a growing body of evidence suggests that the placement process is significantly impacted by restrictive market conditions, which hinder local authorities' ability to meet the standards set by their sufficiency duty (Bach-Mortensen, Murray, et al., 2022; CMA, 2022; MacAlister, 2022).

The role of for-profit companies in the children's social care sector remains a heavily debated question. Existing work generally demonstrates that the for-profit outsourcing of public services does not typically transfer into cost reductions or improved service quality (Petersen et al., 2018). Children's social care is no exception: a recent study found that even though the number of for-profit children's homes has heavily increased in the last 10 years (currently representing more than 80 % of this provision in England), these providers are rated, on average, of worse quality than LA and third sector provision (Bach-Mortensen, Goodair, & Barlow, 2022). Further, both the CMA and the Independent Care Review reports document that large chains of for-profit providers have secured increasing control of the market and evidenced how these companies leveraged this position to maximise profits (CMA, 2022; MacAlister, 2022).

Despite this, expanding the use of the private market continues to be frequently suggested as a solution to the sufficiency crisis (Le Grand, 2007). The CMA produced three recommendations to expand the capacity of children's social care in England. The first two of these suggested a) identifying and removing ‘restrictive’ sectoral regulations and b) changing planning regulations to enable providers to enter the market more easily (CMA, 2022). Implicit in these recommendations is the assumption that greater use and involvement of the for-profit sector will solve the issues caused by the insufficient number of placements. This assumption remains unverified.

To date, there is no evidence on the role of outsourcing in terms of the placement locality and stability among children in care. This is an important research gap, in that placement stability and out-of-area placements are considered key outcomes in the sector. This paper aims to address this gap by using novel harmonized administrative data to conduct the first longitudinal analysis of placement outcomes among children in care in England.

### 1.1 Placement outcomes among children in care

The number of children in need of care being placed outside of their local authority (LA) has been steadily increasing over the past decade, and it was recently estimated that more than 40 % of children in care are placed outside the LA responsible for their care (Foster, 2021). This is a striking development considering that Section 22C of the Children Act 1989 postulates that children should be placed within their home LA to the extent that it is “[…] reasonably practicable”. Moreover, the Sufficiency Duty Guidance highlights the importance of local placements and compels LAs to place children locally (Department for Education, 2010). Yet, there is no detailed or national guidance on when and why to use out of area placements, and it thus left to individual LAs to determine when it is ‘reasonably practicable’ to place children outside their area. Similarly, there is no clear guidance on how to achieve placement stability (defined by the Department for Education as children under 16 living in the same placement for at least 2 years (Department for Education, 2021)), even though the statutory guidance considers this a “[…]critical success factor in relation to better outcomes for looked after children.” (Department for Education, 2021).

Placement stability and locality are considered critical by sector stakeholders due to the long-term risks associated with not achieving placement permanence and placing children at a distance from case workers and their support network (APPG for Looked After Children and Care Leavers, 2022; APPG for Runaway and Missing Children and Adults, 2019; Boddy, 2013; Children's Commissioner, 2019). For example, placement disruptions have been shown to be associated with reduced placement satisfaction among children (Stenason & Romano, 2023) and foster carers (Tonheim & Iversen, 2019), and to be more common among children with trauma (Clark et al., 2020). Similarly, out-of-area placements have been reported to make children more vulnerable to exploitation and grooming (Children's Commissioner, 2019). Furthermore, out-of-area placements may increase costs in terms of travel for social workers, which, in addition, may negatively affect the ability of social workers to monitor the progress and well-being of children.

These risks are further exacerbated by the characteristics of children who experience placement disruptions or are placed out-of-area (APPG for Runaway and Missing Children and Adults, 2019; Children's Commissioner, 2019; Foster, 2021; Mc Grath-Lone et al., 2020). For example, older children with a history of multiple placements, and children with complex needs are more likely to be placed out of area or experience placement disruption (Children's Commissioner, 2019; Mc Grath-Lone et al., 2020; Smith et al., 2022). A 2019 All Party Parliamentary Group (APPG) for Runaway and Missing Children and Adults report found that the number of aggregative missing incidents has increased simultaneously with more children being placed at a distance (APPG for Runaway and Missing Children and Adults, 2019). These issues were raised again in a 2022 report by the APPG for Looked After Children and Care Leavers, which concluded that serious reform “[…] is required to reduce the number of children being placed outside of their local area.” (APPG for Looked After Children and Care Leavers, 2022). However, the report cautioned that “existing commissioning and procurement practices” must be scrutinized before this challenge can be addressed (APPG for Looked After Children and Care Leavers, 2022).

Both the Department for Education and the Competition and Markets Authority consider commissioning and market engagement as the main tools through which to improve outcomes in the sector (CMA, 2022). The last decade has seen the private provision of children's social care grow to such an extent that most LAs are forced to rely on private providers to meet their sufficiency duty to children in need of care. As such, commissioning has increasingly become synonymous with outsourcing children in care to private providers. At the same time, the sufficiency crisis has enabled providers to freely “pick and choose which children they take.” (Ofsted, 2021a). Yet, the connection between commissioning and placement stability and locality has been largely ignored in the academic literature, and it is thus unclear how commissioning influences placement disruptions or out-of-area placements.

### 1.2 Objectives

This paper examines whether the increased outsourcing of children's social care services to the private for-profit sector is influencing placement outcomes. Using data spanning 12 years, we analyse whether children's social care outsourcing is associated to (1) children being placed outside their home LA and (2) aggregate placement stability of children in care at LA level.

## 2 Methods

Our theoretical estimand is the changes to the percentage of children placed close to their local authority given changes in the level of outsourcing to the private sector. By exploiting over-time variation, our analysis investigates whether the increasing use of private provision has resulted in children being placed outside their home local authority, and the extent to which this development has influenced average placement stability.

### 2.1 Data

We use administrative data from the Children looked after (SSDA903) return to analyse placement outcomes of children in care between 2011 and 2022. Children in care are defined in this dataset as any ‘looked after children’ placed in residential or foster and adopted family settings. The data includes information on all children who have been placed in care for any reported reason, including experiences of abuse, neglect, family breakdown and illness. The data collects yearly snapshots on multiple children in care outcomes on the 31st of March each year. It thus enables tracking of placement outcomes, and, in turn, analysis of how these have been impacted by for-profit outsourcing. More details on the full list of data sources and variables can be found in the Supplementary material (see Table A1).

We analyse the impact of outsourcing on two key dependent variables. First, we assess the location of children's social care placements by using the percent of all placements that are located outside the local authority that holds the corporate parentship of a child in care. Location is calculated by the Department for Education (DfE) by analysing the home and placement post codes provided to them by the Local Authorities (Department for Education, 2021).

**Fig. 1:**
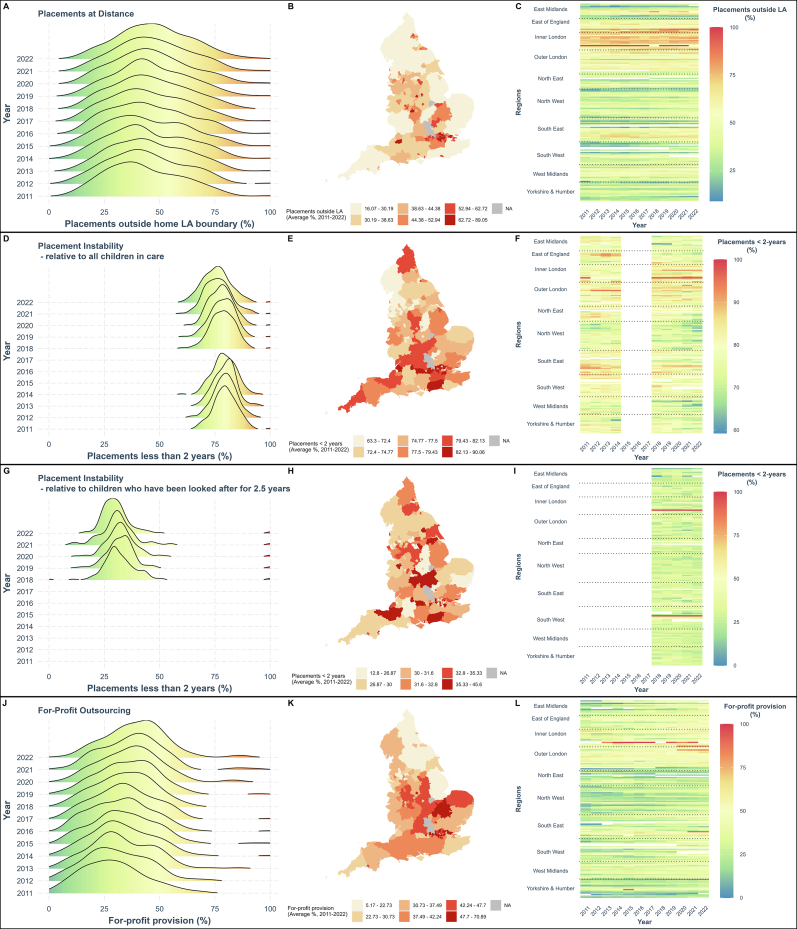
Distributions of local authorities for children in care placement outcomes.

Second, we evaluate the long-term stability of placements, operationalized as the percentage of children in care that have been placed with the same provider for at least 2 years. We construct our own measure of stability, which divides the number of children in stable placements by all children in care within a given Local Authority in order to measure placement stability comparably across time from 2011 to 2022. We support this analysis with a subgroup analysis looking specifically at children who have been in care for long periods of time. Since 2018, data has been available for the percentage of children in care *who have been looked after for at least 2.5 years* who have been placed with the same provider for at least 2 years. We utilise this measure as a sensitivity check, displaying the descriptive statistics in Fig. 1 and reproducing the relationships in the Appendix (Figs. A8 and A9, Table A12). All outcomes are analysed at the local authority level.

### 2.2 Statistical analysis

We present time series regressions, using panel data analysis, on the impact of private for-profit sector outsourcing on placement distance and stability. We run fixed effects regression models, with fixed effects for both units (local authorities) and time (years), on the association between for-profit outsourcing and our outcome variables (out-of-area placements and long-term stability) between 2011 and 2021. These models control for time-invariant confounding by analysing within-unit changes in the explanatory and outcome variables. This approach differs from pooled regression models, in which variables are not structured within units and time. All models control for changes in the proportion of short-term placements, LA funding levels, and child in care characteristics such as gender, ethnicity and the number of children in care.

We run our fixed effects model using covariate balancing with propensity scores based on the number of short-term placements and the levels of LA funding. Covariate balancing is an advanced matching method that has similarities with inverse-probability weighting, which allows us to weight observations based on a characteristic that may be unbalanced according to varying ‘treatment’ levels. Importantly, this technique allows us to adjust for unbalanced treatment groups, even when the ‘treatment’ is a continuous variable (i.e., the percent of placements outsourced to for-profit providers) (Fong et al., 2018). Short-term placements are counted as part of the total number of children in care, even though these constitute a different type of care provision, often referred to as ‘respite care’ or ‘shared care’. The Department for Education (2021) defines short-term placements as “Children cared for in this way normally live at home, but are accommodated by a local authority in a pattern of short periods of care in order to give their parents (or guardians) some ‘respite’ from the normal duties of looking after a child”. We balance our analysis on this variable, because it may confound our results if short-term placements are more frequent among different types of providers.

We further calculate how many additional out-of-area placements can be associated with changes in levels of outsourcing since 2011 by subtracting the marginal effect in our regression models from the observed annual changes in number of placements outside of the LA boundary.

### 2.3 Sensitivity analysis

To confirm the robustness of our findings, we conduct an array of robustness tests and diagnostics, which are detailed in the Supplementary Tables A7–A10, Figs. A3–A7. For example, London's high cost of real estate and service provision and high numbers of children in care, mean that average associations may be driven by the extreme values in London boroughs. To ensure that our analyses are not driven outlier London values, we run the same models removing all London-based LAs from the analysis. Similarly, to avoid extreme values skewing our average associations, we drop each LA in turn to confirm that our results are robust to model specification and not biased by outliers. LAs vary considerably in the size of their jurisdiction. For example the jurisdiction with the most children in care, Lancashire, spans roughly 3000 km^2^, whereas Rutland is almost a tenth of that size. Consequently, we supplemented our main placement distance outcome (outside LA) with analysis of the percentage of children placed within 20 miles from home, which is available over a shorter time span (since 2018) but provides a more precise measure of placement distance. To account for any researcher bias in the selection of covariates we also report specification curves, which test whether the combination of covariates significantly varies the estimates.

## 3 Results

Fig. 1 displays the aggregative changes over time across our placement measures and for-profit outsourcing between 2011 and 2022. More descriptive statistics are available in the Supplementary material (Table A2).

Fig. 1 shows that there is large variation in the proportion of out-of-area placements across local authorities (LAs). Some LAs place almost all children within their boundary, whereas others place all or most children in other LAs. Panel B of Fig. 1 shows that rural LAs have the fewest children placed outside their boundary. Panel C shows that the issue of placing children within the responsible LA is particularly acute for the Inner London region. Across all LAs in our study, there has been a slight increase in the percentage of children being placed at distance: in 2011, 39 % of the children in care on March 31st were placed outside their home LA boundary, and by 2022 this had increased to 43 %.

Most children in care have not been in the same placement continuously for over 2 years (Fig. 1, panels D–F). This is partly because many of the children in care will not have been in care for the 2.5 years necessary to be counted in this measure, or due to placement changes. When looking at long-term stability as the proportion of children who have been looked after for at least 2.5 years, Fig. 1 (panels G–I) shows that 70 % of children in this category have been in the same placement for at 2 years (2022), which is three percentage points less than in 2018 (73 %).

Overall levels of for-profit outsourcing have, on average, increased steadily over this 10-year period. By 2022, 38 % of all children in care are placed with for-profit providers, including children's homes, fostering agencies, or other care providers, which is an increase of 9 percentage points since 2011. However, panels J-L show that there is large variation in how much Local Authorities use the for-profit sector and in how much that has increased (or decreased) for individual LAs. For example, Plymouth placed 88 of their 377 children in care with for-profit companies (23 %) in 2011, whereas in 2022 they reported placing 242 of their 491 children with for-profits (49 %). Other LAs, however, show the opposite trend; for example, Northamptonshire reduced their percent of for-profit outsourced placements from 368 out of 736 in 2011 (50 %) to 429 out of 1144 in 2021 (37 %).

**Fig. 2:**
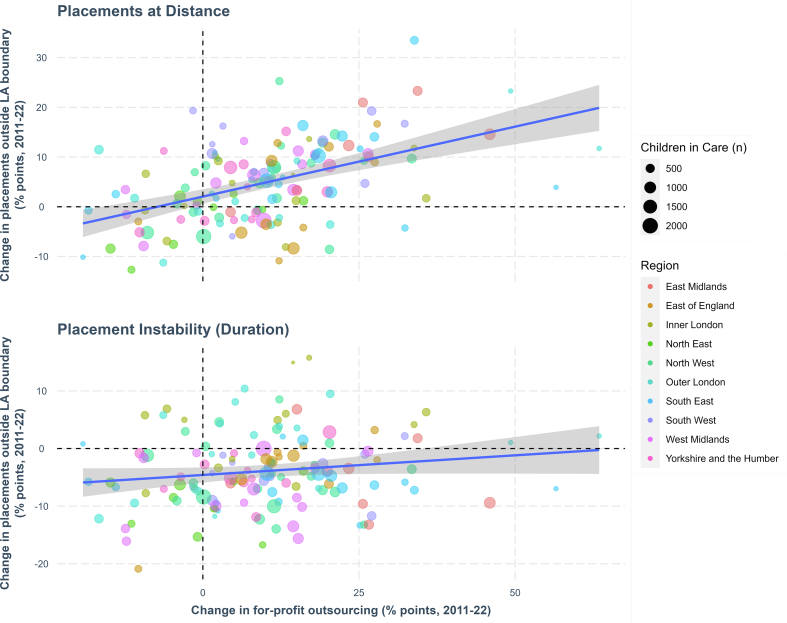
Association between changes in for-profit outsourcing and changes to placement outcome.

Fig. 2 displays the association between changes in for-profit outsourcing and in the placement outcomes over the full time-period. It shows that increases in for-profit outsourcing are positively associated with increasing out-of-area and unstable placements.

Table 1 reports the results of our main statistical analyses. For all our bivariate relationships, we observe that increases in for-profit outsourcing are associated with worse placement outcomes on average. We find that an increase of 1 % of children placed with for-profit providers is associated with an average increase of 0.23 (95 % CI 0.15–0.30; *p* < 0.001) percent more children being placed outside of their home LA, controlling for covariates. This finding is consistent with the more recent placement measure (>20 miles outside the home local authority of children in care) reported in Table A6 of the Supplementary material.

**Table 1:** Relationship between for-profit outsourcing and placement outcomes.

	Two way fixed effects				Covariate balanced using propensity scores		Two way fixed effects				Covariate balanced using propensity scores	
	Placements outside LA [0.95 ci]	*p*-Value	Placements outside LA [0.95 ci]	*p*-Value	Placements outside LA [0.95 ci]	*p*-Value	Placements unstable (%) [0.95 ci]	*p*-Value	Placements unstable (%) [0.95 ci]	*p*-Value	Placements unstable (%) [0.95 ci]	*p*-Value
For-profit outsourcing (%)	0.2743 [0.2087, 0.3399]	<0.0001	0.2347 [0.1654, 0.3041]	<0.0001	0.2261 [0.1532, 0.2990]	<0.0001	0.0903 [0.0310, 0.1496]	0.0046	0.0897 [0.0168, 0.1626]	0.0204	0.0961 [0.0245, 0.1678]	0.0121
Fostering placements (%)			−0.0449 [−0.1507, 0.0609]	0.4081	−0.0493 [−0.1529, 0.0544]	0.3552			−0.0558 [−0.1555, 0.0439]	0.2764	−0.0446 [−0.1378, 0.0486]	0.3528
CIC ethnicity (white, %)			0.0017 [−0.1251, 0.1286]	0.9787	−0.0212 [−0.1430, 0.1006]	0.7345			−0.0924 [−0.2219, 0.0371]	0.1686	−0.0946 [−0.2178, 0.0287]	0.1398
CIC gender (Female, %)			0.1061 [−0.0501, 0.2622]	0.1869	0.0920 [−0.0673, 0.2513]	0.2613			−0.1705 [−0.3325, −0.0086]	0.0428	−0.1764 [−0.3357, −0.0171]	0.0339
CIC (n)			−0.0024 [−0.0086, 0.0037]	0.4438	−0.0030 [−0.0093, 0.0033]	0.3645			0.0052 [−0.0038, 0.0141]	0.2663	0.0065 [−0.0024, 0.0153]	0.1694
Short term only placements (%)			0.0023 [−0.1147, 0.1193]	0.9691	0.0080 [−0.1146, 0.1306]	0.8983			−0.0015 [−0.1216, 0.1186]	0.9808	0.0167 [−0.0915, 0.1249]	0.7645
Children's Social Care Expenditure (£, Ms)			0.1285 [0.0371, 0.2199]	0.0090	0.1432 [0.0512, 0.2351]	0.0054			−0.0577 [−0.1429, 0.0275]	0.1932	−0.0662 [−0.1459, 0.0135]	0.1181

Num.Obs.	1796	1796	1244	1244	1244	1244	1342	1342	932	932	932	932
R2	0.198	0.198	0.209	0.209	0.962	0.962	0.027	0.027	0.070	0.070	0.674	0.674
R2 Adj.	0.116	0.116	0.084	0.084	0.956	0.956	−0.109	−0.109	−0.132	−0.132	0.603	0.603
AIC					6834.9	6834.9					5180.6	5180.6
BIC					7711.5	7711.5					5993.3	5993.3
Log.Lik.					−3246.471	−3246.471					−2422.301	−2422.301
F					161.522	161.522					9.520	9.520
LA fixed effects	Yes	Yes	Yes	Yes	Yes	Yes	Yes	Yes	Yes	Yes	Yes	Yes
Time fixed effects	Yes	Yes	Yes	Yes	Yes	Yes	Yes	Yes	Yes	Yes	Yes	Yes
Clustered standard errors	Yes	Yes	Yes	Yes	Yes	Yes	Yes	Yes	Yes	Yes	Yes	Yes

Table reports results from multivariate longitudinal regression models.

Robust SEs are clustered at LA level and use a bias-reduced linearization estimator (CR2).

Table 1 also reports that an increase of 1 % of for-profit outsourcing is associated with an average increase of 0.10 (95 % CI 0.02–0.17; *p* = 0.01) percent more children being in the same placements for <2 years, controlling for covariates. Both findings are robust to all possible model specifications (see specification curves, Figs. A6–A7 Supplementary material). Moreover, to test whether our findings hold regardless of the construction of our long-term stability variable, we run a regression with the raw number of children in stable, long-term placements as the outcome variable. Here we find that, when controlling for covariates, increases in for-profit outsourcing are still associated with higher rates of long-term placement instability.

To estimate the number of additional children placed out of area due to for-profit outsourcing, we analysed the association of changes in the raw numbers of children placed with private for-profit providers and changes in raw number of children placed outside of the LA boundary on a sub-sample of 138 Local Authorities for which we have data for the full time-period. Controlling for the same covariates as in Table 1, we find that an increase in one placement with a for-profit provider is associated with an increase of 0.27 (95 % CI 0.14–0.40, *p* = 0.0001) placements outside of the LA boundary (see Table A3 in the Supplementary material for full regression results). Based on this coefficient, we calculated how many additional children placed out of LA boundaries can be attributed to the additional use of for-profit outsourcing since 2011. For the 138 LAs used in this analysis, a total of 341,827 children were placed out of area. We calculate that an additional 17,001 (95 % CI 9015-24,987) out-of-area placements between 2011 and 2022 can be attributed to increases in for-profit provision.

**Fig. 3:**
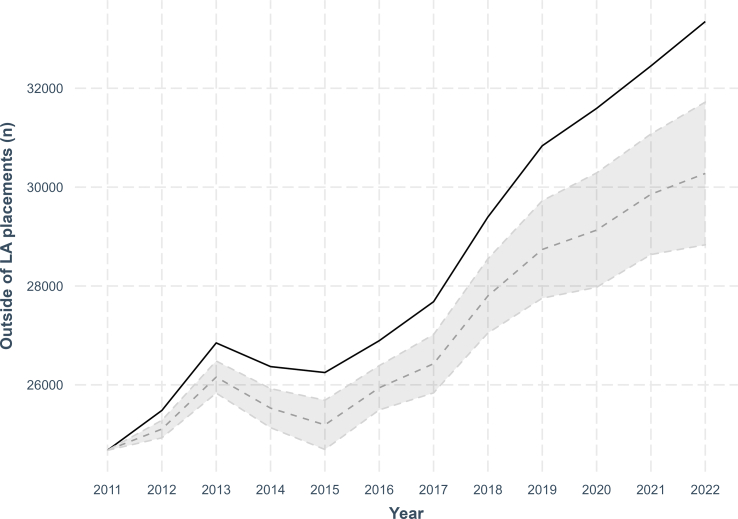
Actual trend in out-of-area placements and expected trend in the absence of changes to for-profit outsourcing 2011–2022. Dashed line represents predicted changes to the number of children in care placed outside their LA boundary had there been no increase in the number of children placed with for-profit providers. Shaded area represents 95 % confidence intervals. Full table of regression used to calculate the average marginal effect and confidence intervals available in Appendix (Table A3).

Fig. 3 visualises the changes in children in care being placed outside of the Local Authority boundary from 2011 to 2022. The dashed line represents the estimated number of children placed of area if there has been no increase in for-profit sector placements, given the associations we observe. Fig. 3 shows that a sizeable amount of the increase in children in placed outside of the LA boundaries may be facilitated by increases in for-profit outsourcing.

## 4 Discussion

Local authority funding cuts and child poverty rates have been shown to be important predictors of key health and social care outcomes (Alexiou et al., 2021; Bennett et al., 2022), but the influence of LA commissioning behavior on such outcomes has not previously been investigated. Our findings demonstrate that for-profit outsourcing is consistently associated with (1) more children being placed outside their home LA and (2) greater long-term placement instability. These results echo previous qualitative research on the shift of out-of-home-care from the public to the private sector, which was not found to improve continuity or consistency among children (Stanley et al., 2013; Stanley et al., 2014).

This paper adds to a growing body of recent research on the negative impacts of for-profit outsourcing in health and social care in England. For-profit outsourcing was recently shown to be associated with higher rates of treatable deaths in the NHS (Goodair & Reeves, 2022), and for-profit ownership has also been found to be associated with lower Care Quality Commission (CQC) ratings in adult residential care (Barron & West, 2017), and lower Ofsted ratings in children's homes (Bach-Mortensen, Goodair, & Barlow, 2022). Despite these findings, the use of private sector services continues to increase in both health and social care.

There are many reasons why local and stable placements are considered key objectives in children's social care. Even though children may need residential or foster care and support, the role of social and family networks matter and are known to be important for children in care (Boddy, 2013; Fallesen, 2014; Mc Grath-Lone et al., 2020). Placing a child out-of-area will be appropriate in some situations, especially for children in need of highly specialised treatment and care. However, it is striking that 43 % of all children in care are currently being placed out of area, even though placement locality is strongly recommended in the official guidance. The premise that placing children outside their local authority is always based on the child's best interests was challenged in a recent report, which reported that out-of-area placements tend to be driven by a lack of suitable local places (Foster, 2021). Placement disruptions, although less prevalent than out-of-area placements, are considered undesirable and placement permanence remains a key objective of out-of-home care for children (Boddy, 2013). The government itself announced that “Moving a child out of placement is a last resort, unless it is in the child's best interests” (UK Parliamentary Debate, 2020).

Placement locality and permanence are both known to facilitate the key purpose of children's social care: to equip children with the skills and support necessary for them to transition back into their home community. Frequent placement disruptions or being placed at distance are known to negatively influence the ability of children to achieve these positive long-term outcomes (Boddy, 2013; Mc Grath-Lone et al., 2020), and has been shown to be particularly detrimental to the educational attainment of children in care (O'Sullivan & Westerman, 2007). Designing commissioning practices that facilitate local, stable, and high-quality care should be a policy and research priority going forward.

These results help document the impact of outsourcing children's social care over time and contribute to our understanding about one of the persistent challenges for children's social care provision in England: ensuring that the children in care are placed in provision that meet their needs and help them transition into adulthood and their community. Over the last decade, regulators, experts, governing authorities, and service users have been stressing the risks associated with the current practice of placing children at great distance from their current support networks and not achieving placement permanence (Children's Commissioner, 2019, Children's Commissioner, 2020; CMA, 2022; MacAlister, 2022). Our analyses show that the ability of LAs to ensure placement stability and consistency for the child remains virtually unchanged over the last decade, and that the LAs that rely most on outsourcing are also the ones with the worst placement outcomes on average.

## 5 Limitations

Four caveats should be considered when interpreting our findings. First, we rely on data from the Children looked after (SSDA903) return, which is known to have missing (often due to confidentiality purposes) and sometimes inconsistent data (Department for Education, 2021) (see page 2 in the Supplementary material for a full discussion of this data). By collecting and analysing data covering 12 years – the full period for which the data is available – across several measures, and by using covariate balancing, we developed robust models on the associations between placement outcomes and outsourcing. The full list of sensitivity checks can be found in the Supplementary material. Second, placement stability and distance may not always represent the best outcomes for children in care. Although long-term placement stability is typically considered a positive outcome, there will be instances where a short-term stay or placement change might be the more appropriate option. Similarly, for children with very complex needs, the most appropriate placement may be located out of area. These outcomes are nonetheless highlighted as key in the sector and are often used as proxies to diagnose the general functioning of children's services. Further, and as discussed above, the prevalence of these outcomes is currently too excessive to be solely driven by the “child's best interests”, especially when considering the sufficiency challenges experienced by most local authorities (Bach-Mortensen, Murray, et al., 2022; CMA, 2022).

Third, our results should be interpreted as associational, given the potential for residual time-variant confounding. However, given that LA services are typically, by definition, placed within a local authority, changes to other provision types should be attributable to changes in the number of children placed outside of LA boundaries. As such, we believe that it is highly plausible that for-profit outsourcing has directly influenced the rates of out of area placements. Fourth, due to the ecological feature of our analysis, our research design is not meant to explore variation on the provider level, and we therefore cannot directly compare placement outcomes between local authority and for-profit providers. Rather, it is our intention with this analysis to evidence the average impact of how the commissioning behavior among local authorities to outsource placements to the for-profit sector is associated with aggregate placement stability and distance among children in care over time. Our findings are nonetheless consistent with the existing provider-level research on outsourcing, showing that the average for-profit provider is rated of lower quality compared to public provision (Bach-Mortensen, Goodair, & Barlow, 2022).

## 6 Conclusion

Despite the numerous independent reviews and investigations, a significant proportion of children are still being placed in unstable or out-of-area care, exposing already vulnerable children to additional risks. Outsourcing and private sector involvement continue to be highlighted as promising avenues for LAs to achieve better outcomes for children. Our analysis shows that for-profit outsourcing is consistently associated with worse placement outcomes among local authorities. This suggests that increasing the already significant proportion of for-profit children's social placements may not be the most effective strategy to improve outcomes in the children's social care sector.

## Declaration of competing interest

None.

## Appendix A Supplementary data

Supplementary material Image 1
