# Supplementary material for: For-profit outsourcing and its effects on placement stability and locality for children in care in England, 2011–2022: A longitudinal ecological analysis
Source: Child Abuse Negl. 2023 Oct;144:106245. doi: 10.1016/j.chiabu.2023.106245 (PMC10933776; doi:10.1016/j.chiabu.2023.106245)
Supplement: Supplementary file 1 — Supplementary material [file mmc1.docx]

**Appendix**

Contents

Data 2

Data cleaning 2

Data sources 2

Table A1 2

Descriptive Table 4

Table A2 4

Association between average changes in for-profit outsourcing and placement outcomes 5

Figure A1 5

Full model specifications 5

Results 6

Raw Changes in for-profit placements and out of boundary placements 6

Table A3 6

Missing children and away from placement incidences 6

Table A4 7

Conditional Growth Curves 7

Table A5 8

Figure A2 9

Alternative outcome measures 9

Table A6 10

Sensitivity Checks 10

Ofsted Ratings 10

Table A7: 10

Table A8: 11

Table A9: 11

Sequentially drop LAs 11

Figure A3 12

Figure A4 12

Nonparametric Covariate Balancing using Propensity Scores 12

Figure A5 13

Removing London 13

Table A10 13

Specification Curves 14

Figure A6 15

Figure A7 15

Regression on raw number of children with stable, long-term placements 16

Table A11 16

Analysis on subgroup of children who have been in care for at least 2.5 years 16

Figure A8 17

Figure A9 17

Table A12 18

# Data

## Data cleaning

The primary data source for this paper comes from the annual SSDA903 returns made by Local Authorities to the department for Education. These were first made in 1992 and are publicly available back to 2011. The returns are presented in one longitudinal dataset between 2018 and 2022, which we have merged with previous annual versions of the data. Analysis is conducted based on Local Authority’s ONS code and changes to LAs is thereby captured in our analysis. The placement variables which have been collected consistently over time are the primary ones analysed in this manuscript to allow for the greatest period analysed. Since 2018, more variables have become available, and we present analysis on those variables in this supplementary material.

It is important to control for levels of expenditure which may confound the relationship between outsourcing and placement outcomes. We therefore merged our dataset with data presented by the Department for Education taken from the Local Authorities S251 returns. This contains data on expenditure on children’s care services. Specifically, we use the total value, again matching by ONS code to prevent any false matches based on name spelling.

We create percentage variables by dividing the reported number of children in our placement outcomes (ie., in for-profit placements or placed outside of LA boundaries) by the total number of children in care in each LA each year. This means that for placement stability – the denominator is all children, even though the measure can only capture children who have been in care for at least 2.5 years.

Since 2015, the SSDA903 returns have included missing and away from placement incidences. These were experimental statistics in 2015 and have since been continued. However, the data has large inconsistencies between LAs, and importantly for our research, across time. This means that changes over time may indicate changes in data collection - not changes in experiences of children in care. If this error is randomly allocated, this should not confound any relationship between outsourcing and placement outcomes. However, this is a strong assumption and we therefore decided to only present the results relating to this variable in the supplementary material and to stress that the data may not be reliable.

## Data sources

### Table A1

Below we present a table of all variables included in our full models with a brief definition and data source and location.

| **Variable Name** | **Definition** | **Data source** | **Raw Data location** |
| --- | --- | --- | --- |
| Placements outside LA | The percent of children in care, at 31^st^ March, in placements located outside the responsible Local Authority. | Department for Education: SSDA903 | *Children looked after in England including adoptions*. Available at [Children looked after in England including adoptions, Reporting Year 2022 – Explore education statistics – GOV.UK (explore-education-statistics.service.gov.uk)](https://explore-education-statistics.service.gov.uk/find-statistics/children-looked-after-in-england-including-adoptions/2022) (accessed 18/11/2022) |
| Placements unstable | The percent of children in care, at 31^st^ March, in placements that have not lasted at least 2 consecutive years. | Department for Education: SSDA903 | *Children looked after in England including adoptions*. Available at [Children looked after in England including adoptions, Reporting Year 2022 – Explore education statistics – GOV.UK (explore-education-statistics.service.gov.uk)](https://explore-education-statistics.service.gov.uk/find-statistics/children-looked-after-in-england-including-adoptions/2022) (accessed 18/11/2022) |
| For-profit Outsourcing (%) | The percent of children in care, at 31^st^ March, in placements provided by private provision (organisations run for profit). | Department for Education: SSDA903 | *Children looked after in England including adoptions*. Available at [Children looked after in England including adoptions, Reporting Year 2022 – Explore education statistics – GOV.UK (explore-education-statistics.service.gov.uk)](https://explore-education-statistics.service.gov.uk/find-statistics/children-looked-after-in-england-including-adoptions/2022) (accessed 18/11/2022) |
| Fostering placements (%) | The percent of children in care, at 31^st^ March, in foster placements | Department for Education: SSDA903 | *Children looked after in England including adoptions*. Available at [Children looked after in England including adoptions, Reporting Year 2022 – Explore education statistics – GOV.UK (explore-education-statistics.service.gov.uk)](https://explore-education-statistics.service.gov.uk/find-statistics/children-looked-after-in-england-including-adoptions/2022) (accessed 18/11/2022) |
| CIC ethnicity (white, %) | The percent of children in care, at 31^st^ March, with white ethnicity. | Department for Education: SSDA903 | *Children looked after in England including adoptions*. Available at [Children looked after in England including adoptions, Reporting Year 2022 – Explore education statistics – GOV.UK (explore-education-statistics.service.gov.uk)](https://explore-education-statistics.service.gov.uk/find-statistics/children-looked-after-in-england-including-adoptions/2022) (accessed 18/11/2022) |
| CIC gender (Female, %) | The percent of children in care, at 31^st^ March, with female gender | Department for Education: SSDA903 | *Children looked after in England including adoptions*. Available at [Children looked after in England including adoptions, Reporting Year 2022 – Explore education statistics – GOV.UK (explore-education-statistics.service.gov.uk)](https://explore-education-statistics.service.gov.uk/find-statistics/children-looked-after-in-england-including-adoptions/2022) (accessed 18/11/2022) |
| CIC (n) | The number of children in care (Looked after Children including foster) at 31^st^ March | Department for Education: SSDA903 | *Children looked after in England including adoptions*. Available at [Children looked after in England including adoptions, Reporting Year 2022 – Explore education statistics – GOV.UK (explore-education-statistics.service.gov.uk)](https://explore-education-statistics.service.gov.uk/find-statistics/children-looked-after-in-england-including-adoptions/2022) (accessed 18/11/2022) |
| Short term only placements (%) | The percent of children in care who were only looked after under a series of short-term placements. | Department for Education: SSDA903 | *Children looked after in England including adoptions*. Available at [Children looked after in England including adoptions, Reporting Year 2022 – Explore education statistics – GOV.UK (explore-education-statistics.service.gov.uk)](https://explore-education-statistics.service.gov.uk/find-statistics/children-looked-after-in-england-including-adoptions/2022) (accessed 18/11/2022) |
| Children's Social Care Expenditure (£, Ms) | Total expenditure on ‘Children Looked After’ in millions of pounds. | Department for Education: S251 returns | *LA and school expenditure.* Available at <https://explore-education-statistics.service.gov.uk/find-statistics/la-and-school-expenditure/2020-21> (accessed 18/11/2022) |

##

## Descriptive Table

Below we present a table of descriptive statistics for all key variables in each given year.

### Table A2

| **Variable** | **N** | **Year** | | **2013, N = 151** | **2014, N = 151** | **2015, N = 151** | **2016, N = 151** | **2017, N = 151** | **2018, N = 154** | **2019, N = 154** | **2020, N = 154** | **2021, N = 154** | **2022, N = 154** |
| --- | --- | --- | --- | --- | --- | --- | --- | --- | --- | --- | --- | --- | --- |
|  |  | **2011, N = 151** | **2012, N = 151** |  |  |  |  |  |  |  |  |  |  |
| **Placements outside LA (%)** | 1,805 |  |  |  |  |  |  |  |  |  |  |  |  |
| N |  | 151 | 151 | 151 | 151 | 151 | 151 | 151 | 150 | 151 | 150 | 150 | 147 |
| Mean (Median) |  | 43 (40) | 44 (41) | 45 (44) | 45 (42) | 45 (43) | 45 (43) | 45 (42) | 46 (44) | 46 (44) | 47 (45) | 47 (45) | 48 (48) |
| Range (SD) |  | 10, 100 (17) | 12, 100 (17) | 13, 100 (17) | 9, 100 (18) | 11, 100 (18) | 15, 100 (17) | 15, 100 (17) | 16, 83 (16) | 14, 100 (17) | 15, 100 (17) | 14, 100 (17) | 13, 94 (16) |
| **For-profit Outsourcing (%)** | 1,797 |  |  |  |  |  |  |  |  |  |  |  |  |
| N |  | 149 | 149 | 149 | 151 | 149 | 151 | 151 | 150 | 151 | 150 | 150 | 147 |
| Mean (Median) |  | 29 (28) | 30 (30) | 33 (32) | 33 (32) | 34 (33) | 33 (33) | 34 (34) | 34 (35) | 36 (37) | 37 (37) | 38 (38) | 39 (39) |
| Range (SD) |  | 0, 70 (14) | 0, 72 (14) | 2, 86 (14) | 1, 100 (15) | 6, 100 (15) | 9, 100 (14) | 7, 100 (14) | 6, 63 (13) | 5, 95 (14) | 6, 85 (14) | 4, 95 (15) | 5, 88 (14) |
| **Placements unstable (%)** | 1,349 |  |  |  |  |  |  |  |  |  |  |  |  |
| N |  | 150 | 150 | 149 | 150 | 0 | 0 | 0 | 151 | 151 | 150 | 150 | 148 |
| Mean (Median) |  | 79.7 (79.7) | 79.5 (79.8) | 79.5 (79.9) | 79.2 (79.0) | NA (NA) | NA (NA) | NA (NA) | 78.5 (78.7) | 78.5 (78.9) | 78.0 (77.9) | 76.4 (76.3) | 75.9 (75.8) |
| Range (SD) |  | 66.9, 100.0 (5.4) | 64.3, 93.6 (5.1) | 67.0, 94.6 (4.9) | 60.0, 94.6 (5.4) | Inf, -Inf (NA) | Inf, -Inf (NA) | Inf, -Inf (NA) | 60.0, 100.0 (5.7) | 60.6, 100.0 (5.6) | 62.9, 100.0 (5.5) | 59.3, 100.0 (6.3) | 60.6, 100.0 (6.4) |
| **CIC (n)** | 1,812 |  |  |  |  |  |  |  |  |  |  |  |  |
| N |  | 151 | 151 | 151 | 151 | 151 | 151 | 151 | 152 | 152 | 150 | 151 | 150 |
| Mean (Median) |  | 434 (377) | 444 (373) | 451 (384) | 456 (385) | 461 (390) | 467 (386) | 481 (409) | 496 (414) | 514 (420) | 533 (450) | 535 (436) | 540 (448) |
| Range (SD) |  | 9, 1,901 (310) | 6, 1,896 (311) | 7, 1,892 (310) | 7, 1,837 (312) | 8, 1,989 (315) | 11, 2,310 (324) | 12, 1,898 (319) | 0, 1,950 (330) | 0, 2,115 (345) | 24, 2,095 (353) | 0, 1,995 (358) | 0, 2,094 (366) |
| **Children's Social Care Expenditure (£, Ms)** | 1,653 |  |  |  |  |  |  |  |  |  |  |  |  |
| N |  | 151 | 151 | 151 | 151 | 149 | 149 | 149 | 151 | 151 | 150 | 150 | 0 |
| Mean (Median) |  | 20 (17) | 20 (17) | 23 (20) | 24 (21) | 25 (22) | 26 (23) | 28 (23) | 29 (24) | 32 (26) | 35 (29) | 37 (30) | NA (NA) |
| Range (SD) |  | 1, 84 (14) | 1, 84 (15) | 0, 94 (16) | 0, 94 (16) | 1, 100 (16) | 1, 100 (17) | 1, 102 (18) | 0, 108 (19) | 0, 116 (21) | 0, 121 (23) | 0, 135 (24) | Inf, -Inf (NA) |
| **Short term only placements (%)** | 1,377 |  |  |  |  |  |  |  |  |  |  |  |  |
| N |  | 143 | 138 | 127 | 110 | 109 | 106 | 107 | 111 | 106 | 108 | 104 | 108 |
| Mean (Median) |  | 10.8 (10.4) | 8.7 (8.4) | 6.4 (5.1) | 4.8 (3.8) | 3.9 (2.6) | 3.2 (2.1) | 2.6 (1.0) | 2.0 (0.0) | 1.7 (0.0) | 1.3 (0.0) | 1.2 (0.0) | 1.1 (0.0) |
| Range (SD) |  | 0.0, 31.7 (6.5) | 0.0, 25.8 (5.9) | 0.0, 25.0 (5.5) | 0.0, 22.3 (4.8) | 0.0, 21.3 (4.3) | 0.0, 18.1 (3.8) | 0.0, 20.1 (3.7) | 0.0, 19.5 (3.4) | 0.0, 17.3 (3.3) | 0.0, 14.4 (2.9) | 0.0, 14.6 (2.9) | 0.0, 12.0 (2.6) |

## Association between average changes in for-profit outsourcing and placement outcomes

In the manuscript (figure 2) we present the association between changes in % point differences in for-profit outsourcing and placement outcomes across the whole time-period. Here were present a similar figure but with average annual changes for each LA across the full time-period.

### Figure A1


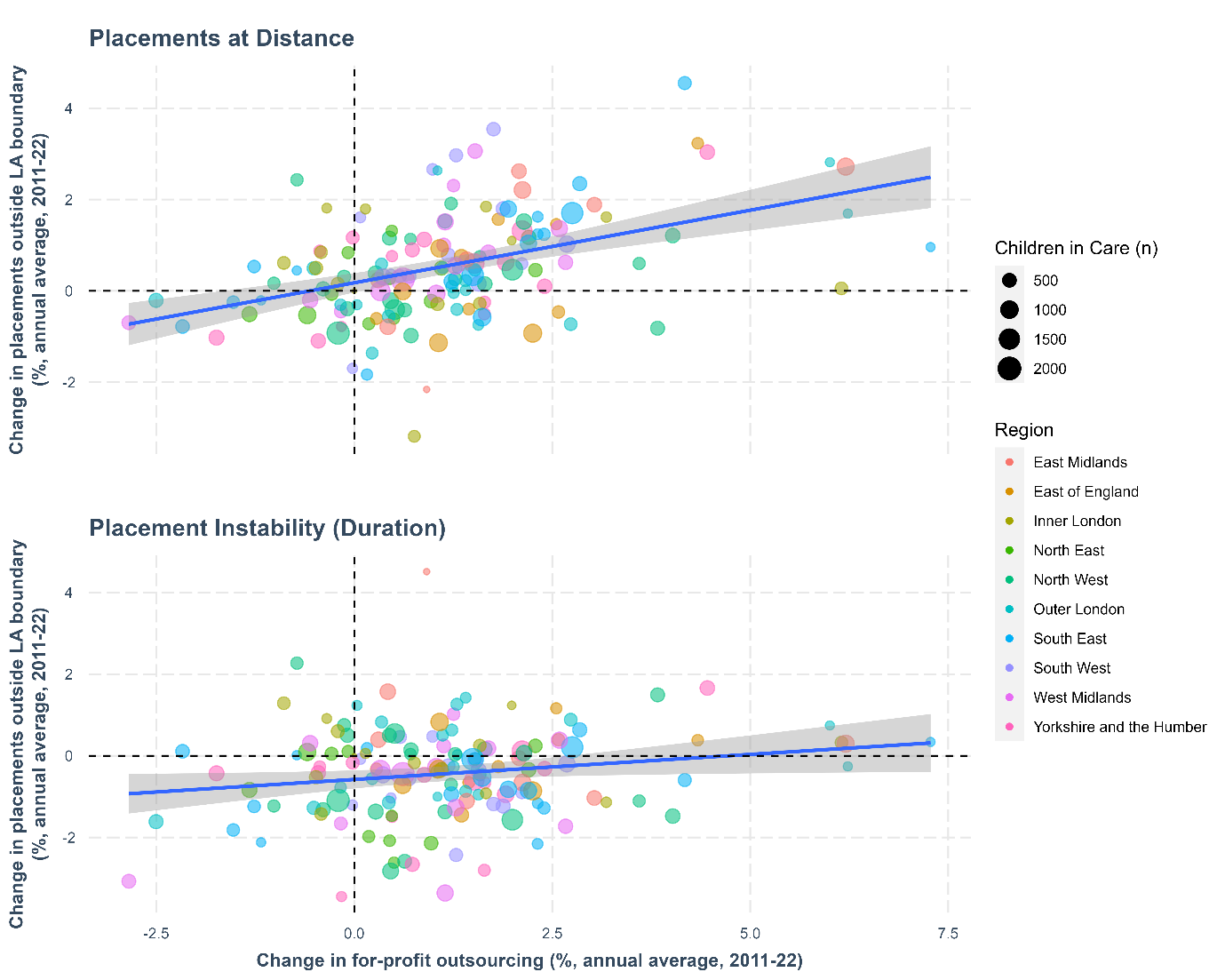


## Full model specifications

Our full TWFE models are fixed effects models with fixed effects for year and LA. The model is:

*PLACEMENT𝑖𝑡* = 𝛽𝑂𝑈𝑇𝑖𝑡 + *𝛽FOSTER𝑖𝑡* + *𝛽ETHNIC𝑖𝑡* + *𝛽FEMALE𝑖𝑡* + *𝛽CIC𝑖𝑡* + *𝛽STP𝑖𝑡* + *𝛽EXPEND𝑖𝑡* + 𝛼𝑖 + 𝜇𝑖𝑡

Where i is a given LA, t is a given year, PLACEMENT is the placement outcome (stability/ location), OUT is for-profit outsourcing, FOSTER is the % of placements in foster care, ETHNIC is the % if children in care who with white ethnicity, FEMALE is the % of children in care with Female Gender, CIC is the number of children in care, STP is the % of placements which are short-term-only placements, EXPEND is the total expenditure on Looked after children’s social care services, ai is the unobserved time-invariant individual effect and uit is the error term.

The remaining models (CBPS) are fixed effects models, where the observations are weighted based on the number of short-term only placements and total children’s social care expenditure in each LA. We use the non-parametric covariate balancing using propensity scores method advocated by Fong, Hazlett and Imai (2018), in which the weights, wi, for observations are (stabilized) inverse generalized propensity score weights. They are specified as:

$$w_{i} = \frac{f (T_{i}^{*} )}{{f (T_{i}^{*} |X_{i}^{*} )}^{'}}$$

T is our treatment of for-profit outsourcing and X is our covariates of number of short-term only placements and total children’s social care expenditure. f (T𝑖 ∗ ) represents the marginal distribution of treatments and f (T𝑖 ∗ |X𝑖 ∗ ) ′ represents the generalised propensity score.

# Results

## Raw Changes in for-profit placements and out of boundary placements

To give a sense of the size of the association and what that means over the period studied, we wanted to show how many additional children’s placements which are located out of the LA boundary can be associated with increases in levels of outsourcing since 2011. To achieve this, we run a regression changing independent variable to the number of for-profit placements (rather than the percent) and the dependent variable to the number of placements outside the LA (rather than the percent). We also remove any LAs for which we do not have 12 years of data. The coefficient of this model is then used to calculate the additional placements out of LA boundaries attributable to increases in for-profit outsourcing and the values represented in figure 3 in the manuscript. Below we present the results of that regression:

### Table A3

|  | **Placements outside LA [.95 ci]** | **p-value** |
| --- | --- | --- |
| Number of For-Profit Placements (n) | 0.2724 [0.1485, 0.3963] | 0.0001 |
| Fostering placements (%) | 0.0840 [-0.5099, 0.6779] | 0.7824 |
| CIC ethnicity (white, %) | 0.0351 [-0.7414, 0.8115] | 0.9298 |
| CIC sex (Female, %) | 0.6628 [-0.0493, 1.3748] | 0.0719 |
| CIC (n) | 0.2724 [0.1718, 0.3729] | 0.0000 |
| Short term only placements (%) | 0.0110 [-0.5807, 0.6027] | 0.9711 |
| Children's Social Care Expenditure (£, Ms) | 0.1047 [-0.9644, 1.1738] | 0.8490 |
| Num.Obs. | 1168 | 1168 |
| R2 | 0.975 | 0.975 |
| R2 Adj. | 0.971 | 0.971 |
| AIC | 10428.6 | 10428.6 |
| BIC | 11218.4 | 11218.4 |
| Log.Lik. | -5058.293 | -5058.293 |
| F | 255.802 | 255.802 |
| LA Fixed Effects | Yes | Yes |
| Time Fixed Effects | Yes | Yes |
| Clustered Standard Errors | Yes | Yes |
| Table reports results from multivariate longitudinal regression models. | | |
| Robust SEs are clustered at LA level and use a bias-reduced linearization estimator (CR2) | | |

## Missing children and away from placement incidences

One key placement outcome for children in care, commissioners, and placement providers is the number of missing and away from placement incidents. See the Data cleaning section of this appendix for a discussion on the caveats of using variable. In short, this outcome is subject to significant measurement error in the data so our results must be considered with caution. Below we present the same regression models with two alternative outcomes: a) the % of children in care who experienced a missing incident during the year and b) the % of children in care who experienced an away from placement incident during the year.

We find that increases in outsourcing are associated with higher levels of missing incidents for children in care. This association is statistically significant at the p<0.05 or p<0.1 level depending on the specification and control variables. We also find a positive association with Away from placement incidents with a similar effect size, but the results are mostly statistically insignificant at the p<0.05 level.

### Table A4

|  | **TWFE** | | **TWFE** | | **CBPS** | | **TWFE** | | **TWFE** | | **CBPS** | |
| --- | --- | --- | --- | --- | --- | --- | --- | --- | --- | --- | --- | --- |
|  | **Missing Incidents [.95 ci]** | **p-value** | **Missing Incidents [.95 ci]** | **p-value** | **Missing Incidents [.95 ci]** | **p-value** | **Away Incidents (%) [.95 ci]** | **p-value** | **Away Incidents (%) [.95 ci]** | **p-value** | **Away Incidents (%) [.95 ci]** | **p-value** |
| For-profit Outsourcing (%) | 0.0346 [0.0037, 0.0655] | 0.0362 | 0.0384 [-0.0030, 0.0798] | 0.0852 | 0.0392 [-0.0042, 0.0827] | 0.0927 | 0.0362 [-0.0024, 0.0748] | 0.0758 | 0.0362 [-0.0185, 0.0910] | 0.2077 | 0.0421 [-0.0134, 0.0976] | 0.1498 |
| Fostering placements (%) |  |  | -0.0561 [-0.1312, 0.0190] | 0.1483 | -0.0732 [-0.1476, 0.0012] | 0.0594 |  |  | -0.0367 [-0.0970, 0.0237] | 0.2403 | -0.0411 [-0.1002, 0.0179] | 0.1802 |
| CIC ethnicity (white, %) |  |  | -0.0721 [-0.1858, 0.0417] | 0.2198 | -0.0634 [-0.1745, 0.0477] | 0.2692 |  |  | -0.1014 [-0.2118, 0.0091] | 0.0779 | -0.0789 [-0.1830, 0.0252] | 0.1445 |
| CIC gender (Female, %) |  |  | 0.0630 [-0.0650, 0.1909] | 0.3380 | 0.0711 [-0.0545, 0.1968] | 0.2714 |  |  | 0.1200 [-0.0111, 0.2512] | 0.0776 | 0.1230 [-0.0005, 0.2465] | 0.0559 |
| CIC (n) |  |  | -0.0047 [-0.0099, 0.0004] | 0.0977 | -0.0042 [-0.0090, 0.0006] | 0.1210 |  |  | -0.0056 [-0.0121, 0.0009] | 0.1249 | -0.0049 [-0.0099, 0.0002] | 0.1002 |
| Short term only placements (%) |  |  | 0.1287 [-0.0345, 0.2920] | 0.1389 | 0.0892 [-0.0886, 0.2669] | 0.3386 |  |  | -0.0153 [-0.1955, 0.1648] | 0.8691 | -0.0322 [-0.2143, 0.1499] | 0.7320 |
| Children's Social Care Expenditure (£, Ms) |  |  | 0.0307 [-0.0447, 0.1062] | 0.4336 | 0.0277 [-0.0497, 0.1050] | 0.4936 |  |  | 0.0104 [-0.0844, 0.1051] | 0.8325 | 0.0103 [-0.0707, 0.0912] | 0.8078 |
| Num.Obs. | 1178 | 1178 | 720 | 720 | 720 | 720 | 1008 | 1008 | 617 | 617 | 617 | 617 |
| R2 | 0.007 | 0.007 | 0.034 | 0.034 | 0.743 | 0.743 | 0.007 | 0.007 | 0.034 | 0.034 | 0.746 | 0.746 |
| R2 Adj. | -0.151 | -0.151 | -0.247 | -0.247 | 0.669 | 0.669 | -0.183 | -0.183 | -0.299 | -0.299 | 0.658 | 0.658 |
| AIC |  |  |  |  | 3420.7 | 3420.7 |  |  |  |  | 2962.0 | 2962.0 |
| BIC |  |  |  |  | 4171.7 | 4171.7 |  |  |  |  | 3669.9 | 3669.9 |
| Log.Lik. |  |  |  |  | -1546.331 | -1546.331 |  |  |  |  | -1320.976 | -1320.976 |
| F |  |  |  |  | 9.961 | 9.961 |  |  |  |  | 8.501 | 8.501 |
| LA Fixed Effects | Yes | Yes | Yes | Yes | Yes | Yes | Yes | Yes | Yes | Yes | Yes | Yes |
| Time Fixed Effects | Yes | Yes | Yes | Yes | Yes | Yes | Yes | Yes | Yes | Yes | Yes | Yes |
| Clustered Standard Errors | Yes | Yes | Yes | Yes | Yes | Yes | Yes | Yes | Yes | Yes | Yes | Yes |
| Table reports results from multivariate longitudinal regression models. | | | | | | | | | | | | |
| Robust SEs are clustered at LA level and use a bias-reduced linearization estimator (CR2) | | | | | | | | | | | | |

## Conditional Growth Curves

We wanted to display how the changes in placement outcomes have developed over time. To do this we run random effects models allowing the slope for time to vary according to levels of outsourcing. Without the interaction effects, it is clear that LAs with higher levels of outsourcing have worse placement outcomes. When we interact time with outsourcing, we see that for placement stability, changes are improving quicker for LAs with lower levels of outsourcing.

### Table A5

|  | **Placements outside LA** | **Placements outside LA** | **Placements unstable (%)** | **Placements unstable (%)** |
| --- | --- | --- | --- | --- |
| Time | 0.148*** | 0.214** | -0.387*** | -0.598*** |
|  | (0.028) | (0.071) | (0.029) | (0.080) |
| For-profit Outsourcing (%) | 0.289*** | 4.147 | 0.103*** | -12.755** |
|  | (0.013) | (3.784) | (0.013) | (4.214) |
| Time × For-profit Outsourcing (%) |  | -0.002 |  | 0.006** |
|  |  | (0.002) |  | (0.002) |
| Num.Obs. | 1796 | 1796 | 1342 | 1342 |
| R2 Marg. | 0.078 | 0.082 | 0.190 | 0.194 |
| R2 Cond. | 0.943 | 0.939 |  |  |
| AIC | 10614.3 | 10627.1 | 7781.0 | 7762.5 |
| BIC | 10652.7 | 10671.0 | 7817.4 | 7804.1 |
| ICC | 0.9 | 0.9 |  |  |
| RMSE | 3.53 | 3.53 | 3.63 | 3.58 |
| aicc | 10614.329 | 10627.159 | 7781.066 | 7762.636 |
| + p < 0.1, * p < 0.05, ** p < 0.01, *** p < 0.001 | | | | |

To visualise the conditional growth curve, we present predicted probabilities from the model for the highest and lowest quartile of outsourcers across 11 years of observations. It shows that higher outsourcing LAs are, on average, predicted to place more children in instable and distant placements. For placement stability, it also shows that the change over time is improving more quickly for low outsourcing LAs compared to high outsourcing LAs

### Figure A2


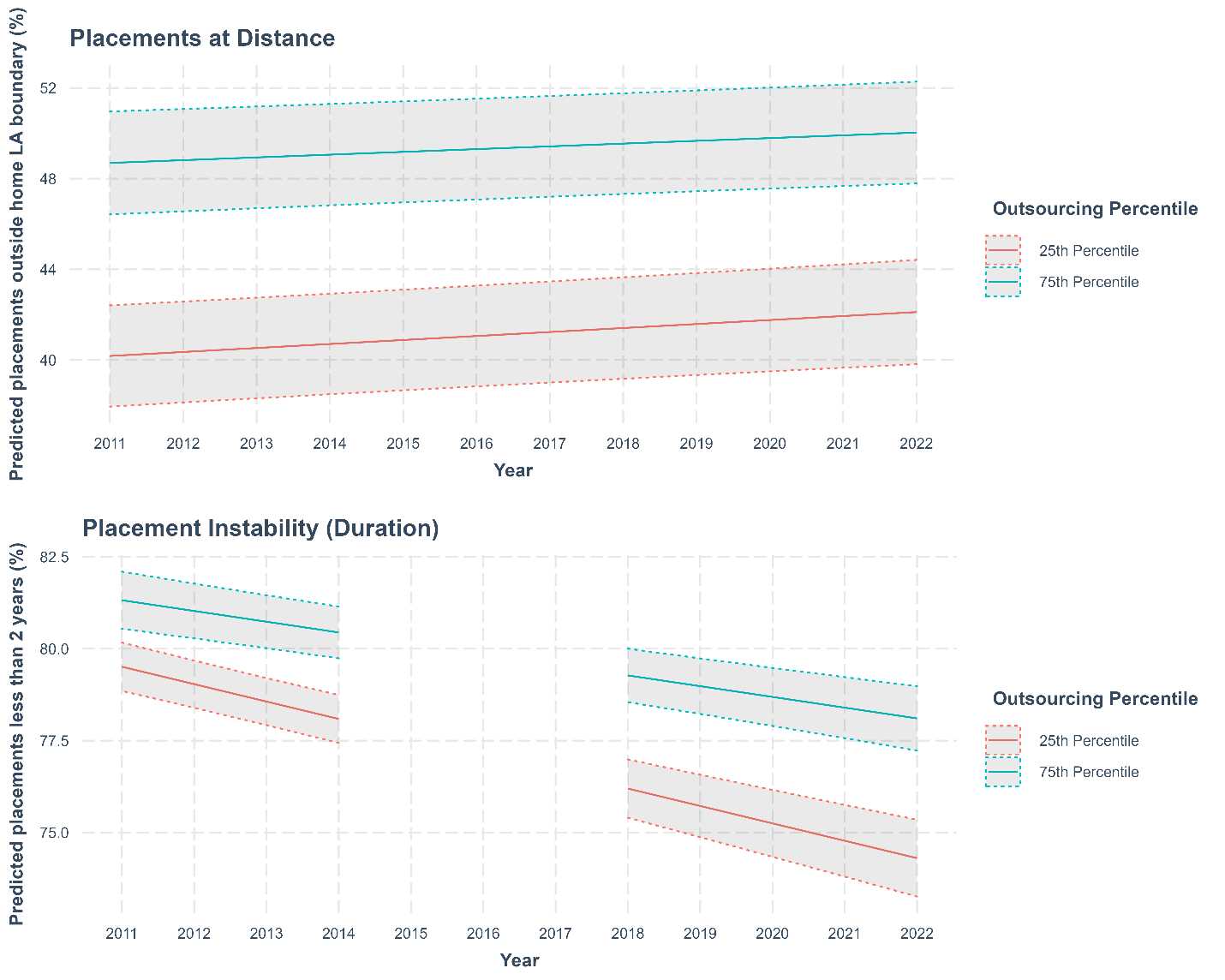


## Alternative outcome measures

Our main findings are estimated based on the two main measures of placement stability and location, which have been collected since 2011. In more recent data releases, other measures have also been reported. These include whether or not the child’s placement is within 20 miles of their home. This is clearly an improved measure of whether or not the child in care can access their existing social support networks, given that being outside the LA boundary could still be very close – particularly in London for example where the LAs are geographically very small. When we use this measure, we produce similar results compared to using whether the placement is inside or outside the LA boundary – i.e., finding that more for-profit outsourcing is associated with more children being placed over 20 miles from home.

More recent data also includes a measure of whether children in care have experienced more than 3 placements during the year. This may not be a better measure of stability than the percent of children not in placements for over 2 years, but it captures an alternative aspect of instability. Interestingly, we find a high effect size with outsourcing being associated with even more of this measure of instability. However, given the sample size and variation in this association, it is statistically insignificant in our models.

### Table A6

|  | **TWFE** | | **TWFE** | | **CBPS** | | **TWFE** | | **TWFE** | | **CBPS** | |
| --- | --- | --- | --- | --- | --- | --- | --- | --- | --- | --- | --- | --- |
|  | **Placements over 20 miles [.95 ci]** | **p-value** | **Placements over 20 miles [.95 ci]** | **p-value** | **Placements over 20 miles [.95 ci]** | **p-value** | **More than 3 placements (%) [.95 ci]** | **p-value** | **More than 3 placements (%) [.95 ci]** | **p-value** | **More than 3 placements (%) [.95 ci]** | **p-value** |
| For-profit Outsourcing (%) | 0.1483 [0.0988, 0.1977] | 0.0000 | 0.1024 [0.0573, 0.1475] | 0.0023 | 0.1009 [0.0562, 0.1456] | 0.0030 | 0.0363 [-0.0122, 0.0847] | 0.1671 | 0.0319 [-0.0264, 0.0903] | 0.3135 | 0.0308 [-0.0310, 0.0926] | 0.3579 |
| Fostering placements (%) |  |  | -0.0208 [-0.1173, 0.0757] | 0.6754 | -0.0170 [-0.1126, 0.0785] | 0.7295 |  |  | -0.0444 [-0.1127, 0.0239] | 0.2096 | -0.0495 [-0.1209, 0.0218] | 0.1826 |
| CIC ethnicity (white, %) |  |  | 0.2258 [0.0993, 0.3522] | 0.0012 | 0.2149 [0.0880, 0.3417] | 0.0020 |  |  | 0.0532 [-0.0460, 0.1523] | 0.2997 | 0.0494 [-0.0552, 0.1541] | 0.3600 |
| CIC gender (Female, %) |  |  | -0.0594 [-0.2006, 0.0819] | 0.4155 | -0.0721 [-0.2054, 0.0612] | 0.2966 |  |  | 0.0210 [-0.0857, 0.1278] | 0.7012 | 0.0182 [-0.0894, 0.1259] | 0.7421 |
| CIC (n) |  |  | -0.0022 [-0.0093, 0.0050] | 0.5582 | -0.0034 [-0.0125, 0.0056] | 0.4827 |  |  | 0.0076 [0.0013, 0.0138] | 0.0258 | 0.0062 [-0.0005, 0.0130] | 0.1128 |
| Short term only placements (%) |  |  | -0.1750 [-0.5097, 0.1596] | 0.3251 | -0.1808 [-0.5264, 0.1647] | 0.3217 |  |  | 0.1859 [-0.0625, 0.4343] | 0.1635 | 0.1476 [-0.1250, 0.4201] | 0.3027 |
| Children's Social Care Expenditure (£, Ms) |  |  | 0.0891 [0.0132, 0.1651] | 0.0295 | 0.0780 [0.0013, 0.1546] | 0.0976 |  |  | -0.0206 [-0.0909, 0.0498] | 0.5702 | -0.0146 [-0.0857, 0.0564] | 0.6990 |
| Num.Obs. | 661 | 661 | 369 | 369 | 369 | 369 | 737 | 737 | 418 | 418 | 418 | 418 |
| R2 | 0.106 | 0.106 | 0.175 | 0.175 | 0.976 | 0.976 | 0.013 | 0.013 | 0.064 | 0.064 | 0.641 | 0.641 |
| R2 Adj. | -0.166 | -0.166 | -0.297 | -0.297 | 0.962 | 0.962 | -0.252 | -0.252 | -0.436 | -0.436 | 0.449 | 0.449 |
| AIC |  |  |  |  | 1539.5 | 1539.5 |  |  |  |  | 1625.6 | 1625.6 |
| BIC |  |  |  |  | 2071.3 | 2071.3 |  |  |  |  | 2218.8 | 2218.8 |
| Log.Lik. |  |  |  |  | -633.729 | -633.729 |  |  |  |  | -665.778 | -665.778 |
| F |  |  |  |  | 70.652 | 70.652 |  |  |  |  | 3.348 | 3.348 |
| LA Fixed Effects | Yes | Yes | Yes | Yes | Yes | Yes | Yes | Yes | Yes | Yes | Yes | Yes |
| Time Fixed Effects | Yes | Yes | Yes | Yes | Yes | Yes | Yes | Yes | Yes | Yes | Yes | Yes |
| Clustered Standard Errors | Yes | Yes | Yes | Yes | Yes | Yes | Yes | Yes | Yes | Yes | Yes | Yes |
| Table reports results from multivariate longitudinal regression models. | | | | | | | | | | | | |
| Robust SEs are clustered at LA level and use a bias-reduced linearization estimator (CR2) | | | | | | | | | | | | |

# Sensitivity Checks

## Ofsted Ratings

One of the main contributions of this paper is that we analyse variation in other outcomes relating to placement quality than Ofsted ratings. To test if Ofsted ratings reflect some of these outcomes, we present bivariate regression results comparing our outcomes with the most recent LA inspection results. Due to LAs being inspected very infrequently, we have a very small n and only one observation per LA.

### Table A7:

|  | **Overall Effectiveness** | **Effectiveness of Leaders** | **Experiences of children who need protection** | **Experiences and progress of children** |
| --- | --- | --- | --- | --- |
|  | **(1)** | **(2)** | **(3)** | **(4)** |
| Placements Outside LA (%) | 0.968* | 0.970* | 0.974+ | 0.972* |
|  | (0.013) | (0.013) | (0.013) | (0.014) |
| Num.Obs. | 145 | 145 | 145 | 145 |
| AIC | 368.4 | 367.1 | 330.3 | 334.2 |
| BIC | 407.1 | 405.8 | 369.0 | 372.9 |
| RMSE | 2.34 | 2.47 | 2.14 | 2.31 |
| Region Fixed Effects | Yes | Yes | Yes | Yes |
| + p < 0.1, * p < 0.05, ** p < 0.01, *** p < 0.001 | | | | |

### Table A8:

|  | **Overall Effectiveness** | **Effectiveness of Leaders** | **Experiences of children who need protection** | **Experiences and progress of children** |
| --- | --- | --- | --- | --- |
|  | **(1)** | **(2)** | **(3)** | **(4)** |
| Placements Unstable (%) | 1.020 | 1.034 | 1.004 | 1.027 |
|  | (0.040) | (0.041) | (0.040) | (0.042) |
| Num.Obs. | 106 | 106 | 106 | 106 |
| AIC | 292.6 | 288.3 | 265.9 | 265.6 |
| BIC | 327.2 | 322.9 | 300.5 | 300.2 |
| RMSE | 2.41 | 2.51 | 2.22 | 2.37 |
| Region Fixed Effects | Yes | Yes | Yes | Yes |
| + p < 0.1, * p < 0.05, ** p < 0.01, *** p < 0.001 | | | | |

### Table A9:

|  | **Overall Effectiveness** | **Effectiveness of Leaders** | **Experiences of children who need protection** | **Experiences and progress of children** |
| --- | --- | --- | --- | --- |
|  | **(1)** | **(2)** | **(3)** | **(4)** |
| Missing Incidences (%) | 0.985 | 0.994 | 1.031 | 0.969 |
|  | (0.051) | (0.053) | (0.055) | (0.053) |
| Num.Obs. | 142 | 142 | 142 | 142 |
| AIC | 366.2 | 364.2 | 330.2 | 329.5 |
| BIC | 404.6 | 402.6 | 368.7 | 367.9 |
| RMSE | 2.35 | 2.48 | 2.15 | 2.31 |
| Region Fixed Effects | Yes | Yes | Yes | Yes |
| + p < 0.1, * p < 0.05, ** p < 0.01, *** p < 0.001 | | | | |

## Influence of individual local authorities

We present models reporting on the average association within each LA over time. Consequently, it may be the case that the average association could be driven by a significant outlier. To test for this, we report results from the same models in which we sequentially drop each LA from the data. In figures A1 and A2, we present the coefficient and p-value for our full fixed-effects models removing each LA one at a time – with figure A1 showing results for placement location and figure A2 reporting placement stability. We find that the coefficient size varies minimally (with a range of 0.02 for both measures), and that all results remain statistically significant at the p<0.05 level.

### Figure A3


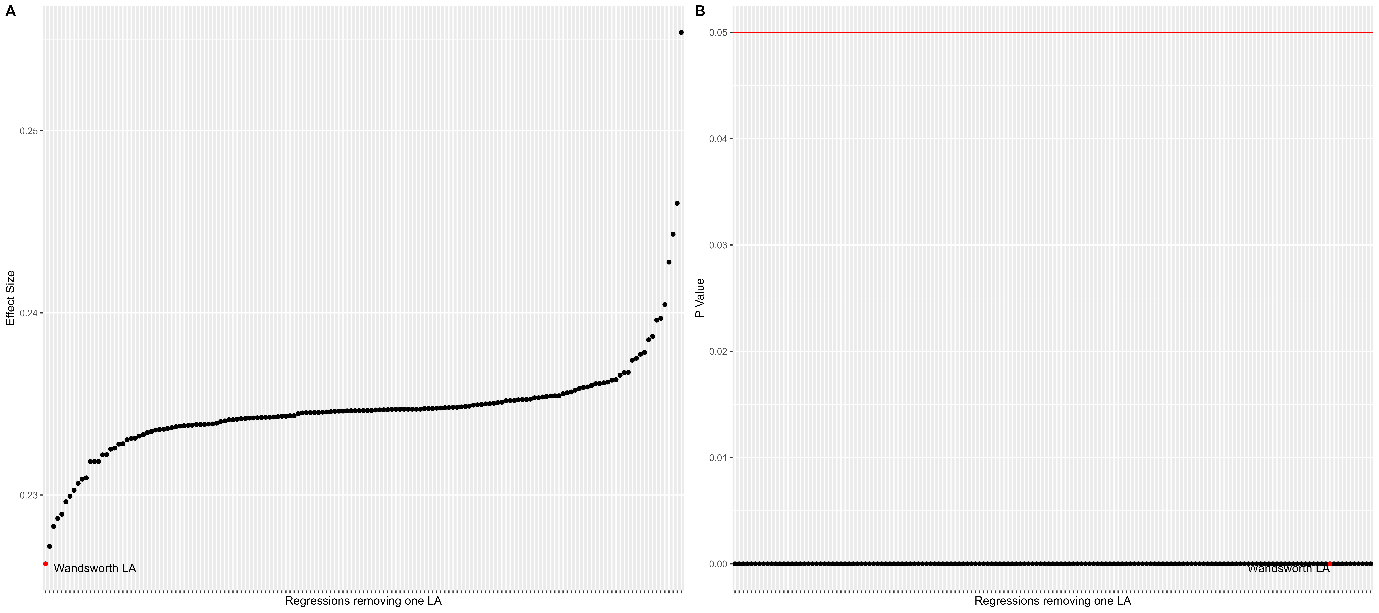


### Figure A4


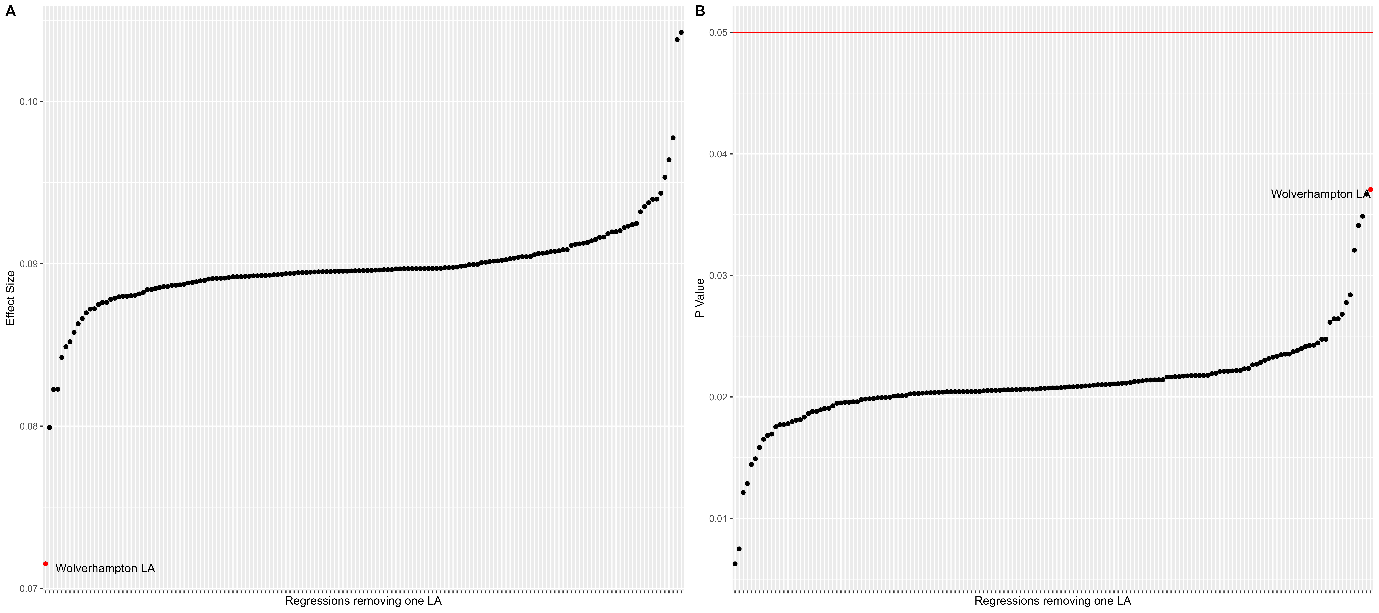


## Nonparametric Covariate Balancing using Propensity Scores

In our manuscript, we present two models which include covariate balancing using propensity scores. The idea of these models is to balance the sample based on key variables – as would be done were analysis to be done on a treatment and control group – but with a continuous ‘treatment’ of for-profit outsourcing.

We conduct analyses using the CBPS package in R. Covariate balancing is an advanced matching method which can weight values to balance the model, accounting for differences in observations according to their value of a continuous treatment variable - in this case for-profit outsourcing.

We balance our sample on the number of children in short-term-only placements and on the total LA expenditure on Looked After Children’s services. Below we present the balance plots of how the weights are applied to both variables we are balancing on. ‘Treat’ refers to the ‘treatment’ of for-profit outsourcing (%).

The red line represents a linear fit and the blue line is a loess fit. The horizontal black line represents the unweighted treatment mean and the vertical black line represents the unweighted outcome mean. Balance is indicated by the flatness of both fit lines and whether they pass through the intersection of the two black reference lines so figure 5 shows there is some residual imbalance in the short term weights when using a loess fit but very little in the other measures.

### Figure A5


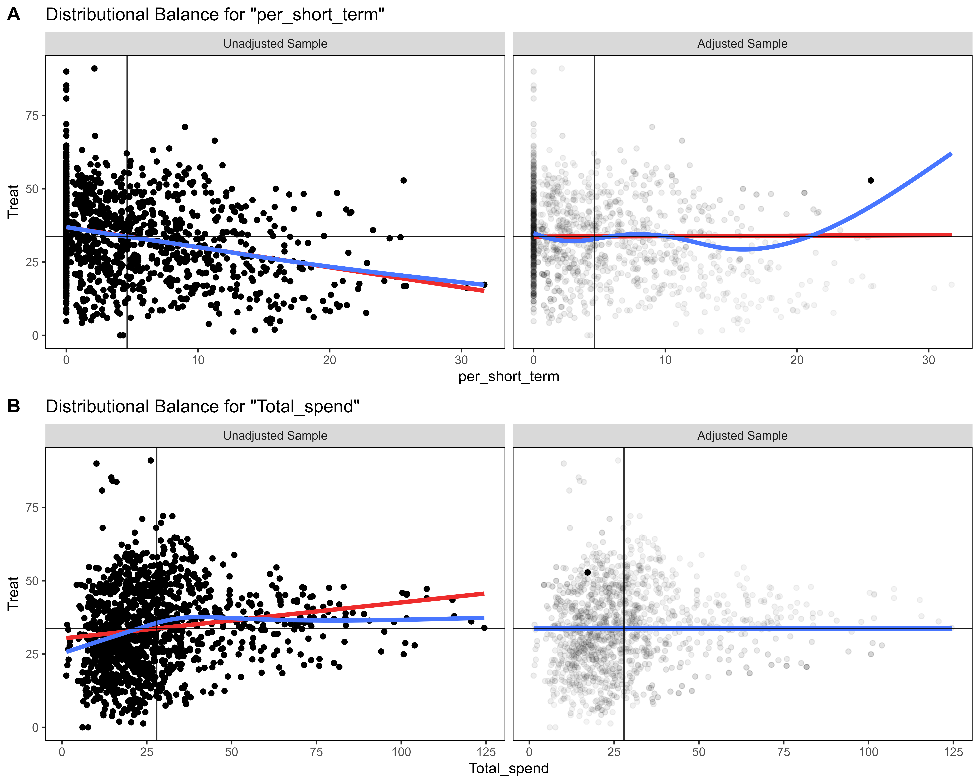


## Removing London

In our manuscript, we include all Local Authorities who present data which is not supressed due to small numbers or confidentiality concerns. This is our full sample, because we are interested in average associations in England. We have already tested for whether a single outlier might explain our results by dropping each LA sequentially (figures A1 - A2). However, we also test whether the relationships we observe are primarily driven by London LAs. London has been reported to experience severe challenges in finding placement sufficiency and our data shows that the LAs in London have much higher rates of children being placed in other LA boundaries.

The table below removes London from the results and finds very similar results to our main findings. When we weight our observations for expenditure and short term placements, our finding for placement location has a smaller effect size and the statistical significance level is p=0.07. This suggess that the relationship between outsourcing and placement location might be particularly strong in London.

### Table A10

|  | **TWFE** | | **TWFE** | | **CBPS** | | **TWFE** | | **TWFE** | | **CBPS** | |
| --- | --- | --- | --- | --- | --- | --- | --- | --- | --- | --- | --- | --- |
|  | **Placements outside LA [.95 ci]** | **p-value** | **Placements outside LA [.95 ci]** | **p-value** | **Placements outside LA [.95 ci]** | **p-value** | **Placements unstable (%) [.95 ci]** | **p-value** | **Placements unstable (%) [.95 ci]** | **p-value** | **Placements unstable (%) [.95 ci]** | **p-value** |
| For-profit Outsourcing (%) | 0.3230 [0.2336, 0.4123] | 0.0000 | 0.2682 [0.1741, 0.3622] | 0.0000 | 0.1708 [0.0079, 0.3337] | 0.0758 | 0.0861 [0.0167, 0.1554] | 0.0189 | 0.1031 [0.0157, 0.1905] | 0.0255 | 0.1033 [0.0246, 0.1819] | 0.0289 |
| Fostering placements (%) |  |  | -0.0593 [-0.1777, 0.0590] | 0.3297 | -0.0809 [-0.2133, 0.0515] | 0.2393 |  |  | -0.0252 [-0.1329, 0.0824] | 0.6478 | 0.0103 [-0.0933, 0.1139] | 0.8465 |
| CIC ethnicity (white, %) |  |  | 0.0409 [-0.1062, 0.1879] | 0.5885 | 0.0970 [-0.1330, 0.3269] | 0.4169 |  |  | -0.0965 [-0.2505, 0.0575] | 0.2257 | -0.0671 [-0.2204, 0.0863] | 0.4009 |
| CIC gender (Female, %) |  |  | 0.0759 [-0.1113, 0.2632] | 0.4299 | 0.0015 [-0.2436, 0.2466] | 0.9905 |  |  | -0.0478 [-0.2584, 0.1628] | 0.6583 | -0.0471 [-0.2541, 0.1599] | 0.6584 |
| CIC (n) |  |  | -0.0024 [-0.0088, 0.0040] | 0.4634 | -0.0046 [-0.0116, 0.0025] | 0.2379 |  |  | 0.0067 [-0.0038, 0.0172] | 0.2212 | 0.0074 [-0.0022, 0.0170] | 0.1722 |
| Short term only placements (%) |  |  | 0.0462 [-0.0820, 0.1744] | 0.4834 | 0.0586 [-0.0780, 0.1952] | 0.4071 |  |  | -0.0911 [-0.2222, 0.0399] | 0.1798 | -0.0732 [-0.1953, 0.0489] | 0.2488 |
| Children's Social Care Expenditure (£, Ms) |  |  | 0.1169 [0.0047, 0.2292] | 0.0497 | 0.2157 [0.0615, 0.3699] | 0.0367 |  |  | -0.0343 [-0.1409, 0.0722] | 0.5320 | -0.0173 [-0.1154, 0.0808] | 0.7430 |
| Num.Obs. | 1404 | 1404 | 976 | 976 | 976 | 976 | 1051 | 1051 | 723 | 723 | 723 | 723 |
| R2 | 0.241 | 0.241 | 0.237 | 0.237 | 0.952 | 0.952 | 0.023 | 0.023 | 0.059 | 0.059 | 0.755 | 0.755 |
| R2 Adj. | 0.161 | 0.161 | 0.112 | 0.112 | 0.944 | 0.944 | -0.116 | -0.116 | -0.155 | -0.155 | 0.699 | 0.699 |
| AIC |  |  |  |  | 5414.2 | 5414.2 |  |  |  |  | 3976.3 | 3976.3 |
| BIC |  |  |  |  | 6093.0 | 6093.0 |  |  |  |  | 4599.7 | 4599.7 |
| Log.Lik. |  |  |  |  | -2568.080 | -2568.080 |  |  |  |  | -1852.165 | -1852.165 |
| F |  |  |  |  | 120.675 | 120.675 |  |  |  |  | 13.516 | 13.516 |
| LA Fixed Effects | Yes | Yes | Yes | Yes | Yes | Yes | Yes | Yes | Yes | Yes | Yes | Yes |
| Time Fixed Effects | Yes | Yes | Yes | Yes | Yes | Yes | Yes | Yes | Yes | Yes | Yes | Yes |
| Clustered Standard Errors | Yes | Yes | Yes | Yes | Yes | Yes | Yes | Yes | Yes | Yes | Yes | Yes |
| Table reports results from multivariate longitudinal regression models. | | | | | | | | | | | | |
| Robust SEs are clustered at LA level and use a bias-reduced linearization estimator (CR2) | | | | | | | | | | | | |

## Specification Curves

One way to test for the sensitivity of our results is to run the full TWFE models with all possible combinations of covariates. This will show if any one covariate is important in changing the effect size and significance of the key relationships between outsourcing and placement outcomes. This is also a check against ‘researcher degrees of freedom’, and to ensure that covariates have not been selectively chosen to induce a certain result. Below we present specification curves for placement distance (A4) and stability (A5). Both location and stability return statistically significant results for any possible combination of covariates.

### Figure A6


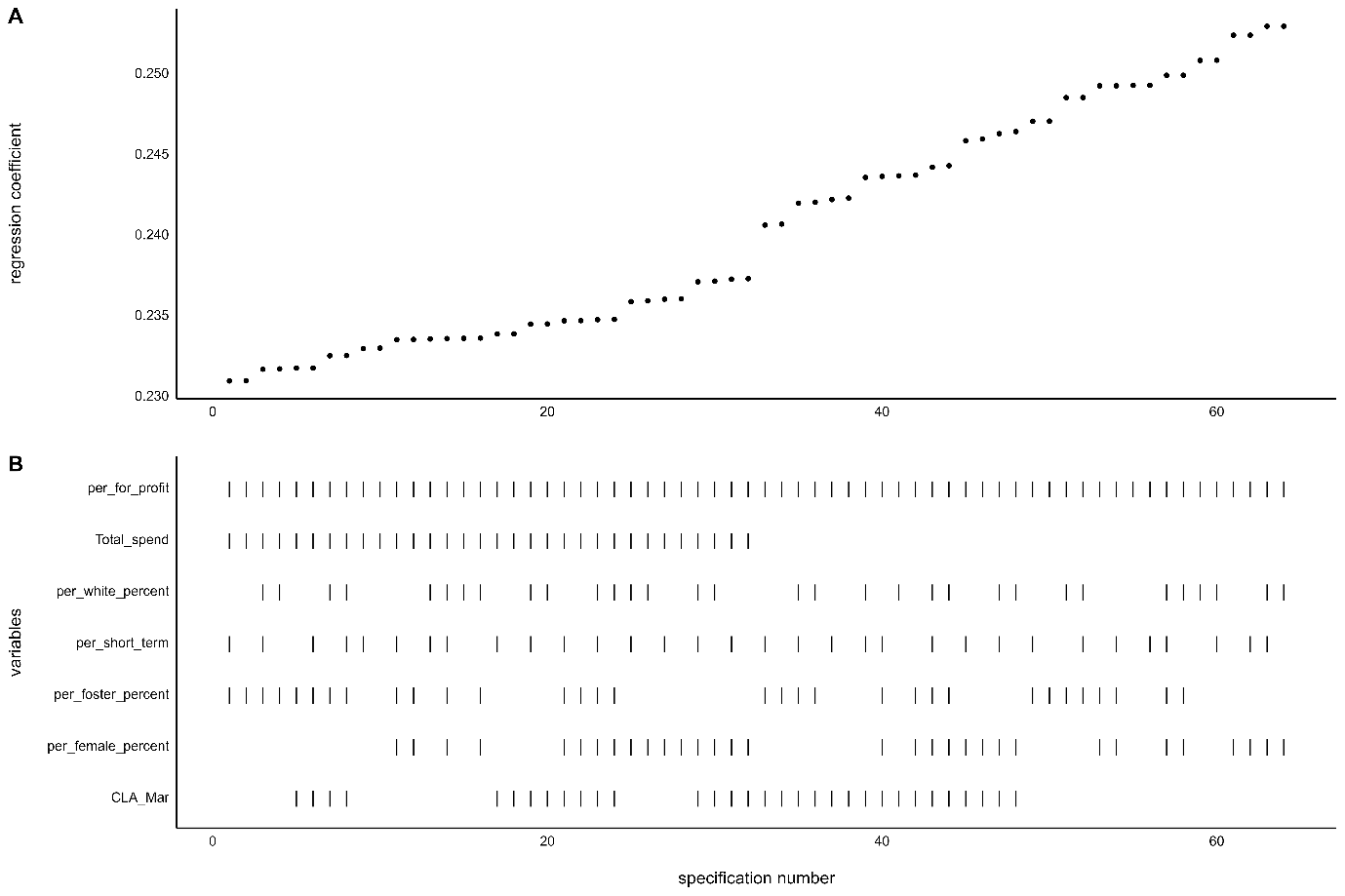


### Figure A7


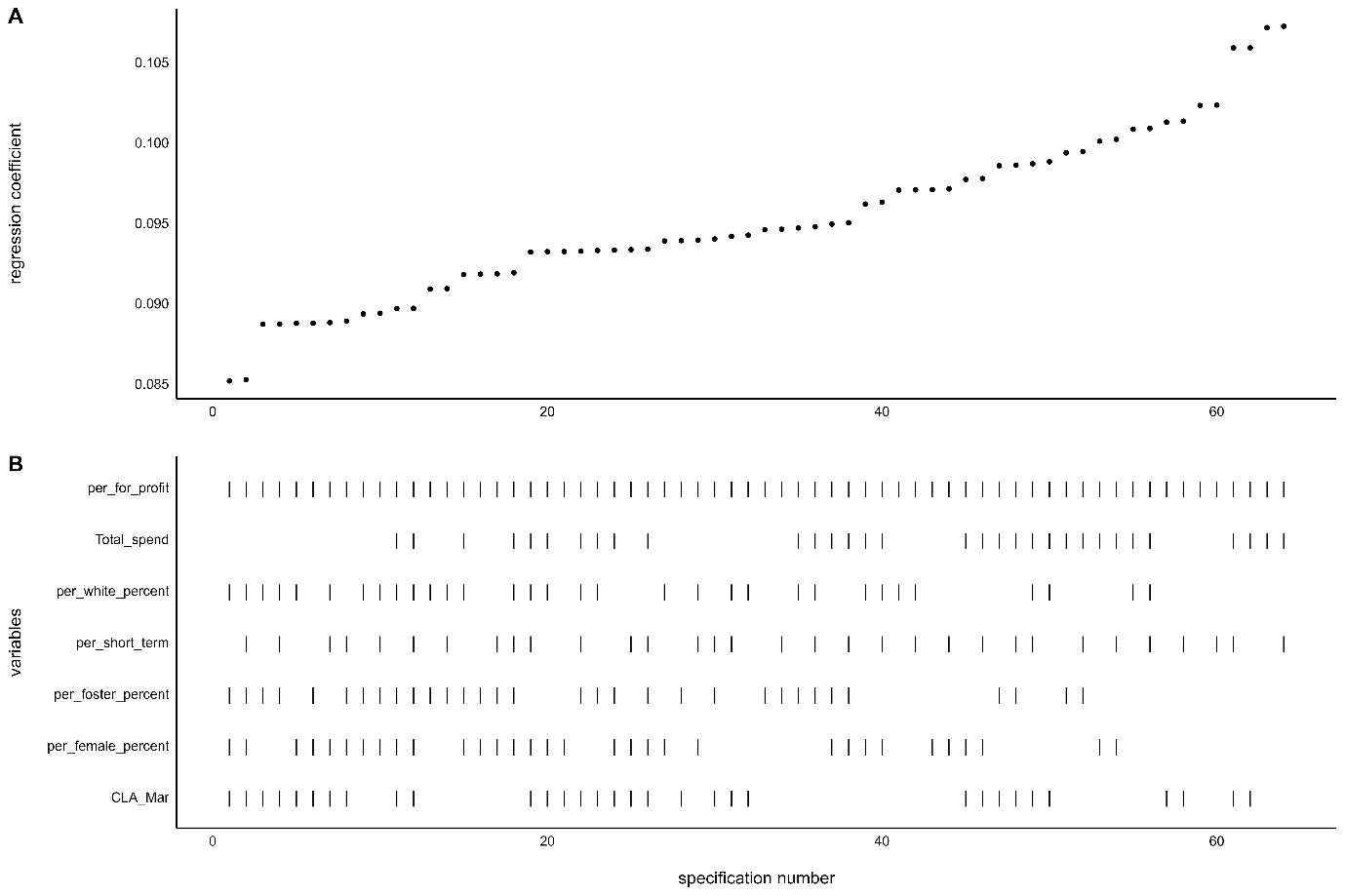


## Regression on raw number of children with stable, long-term placements

Our constructed measure of placement stability is the percent of all children in care with long-term stable placements. To check whether this measure is influenced by the denominator used, we here run the regression on the raw number of children placed in stable placements. After controlling for covariates, we find an increase in 1% point of for-profit outsourcing is associated with an average 0.5 (95% CI 0.2-0.9) fewer children in stable placements.

### Table A11

|  | **TWFE** | **TWFE** | **TWFE** | **TWFE** | **CBPS** | **CBPS** |
| --- | --- | --- | --- | --- | --- | --- |
|  | **Children in placements for over 2 years (n) [.95 ci]** | **p-value** | **Children in placements for over 2 years (n) [.95 ci]** | **p-value** | **Children in placements for over 2 years (n) [.95 ci]** | **p-value** |
| For-profit Outsourcing (%) | -0.0484 [-0.4230, 0.3262] | 0.8013 | -0.4931 [-0.8648, -0.1214] | 0.0129 | -0.5290 [-0.8905, -0.1674] | 0.0066 |
| Fostering placements (%) |  |  | 0.2323 [-0.2818, 0.7463] | 0.3790 | 0.1630 [-0.3428, 0.6689] | 0.5304 |
| CIC ethnicity (white, %) |  |  | 0.3688 [-0.2512, 0.9887] | 0.2497 | 0.4045 [-0.2082, 1.0171] | 0.2025 |
| CIC gender (Female, %) |  |  | 0.3423 [-0.2605, 0.9451] | 0.2695 | 0.4078 [-0.1822, 0.9977] | 0.1805 |
| CIC (n) |  |  | 0.1787 [0.1100, 0.2474] | 0.0000 | 0.1578 [0.0808, 0.2347] | 0.0009 |
| Short term only placements (%) |  |  | -0.0619 [-0.5888, 0.4650] | 0.8187 | -0.0811 [-0.5931, 0.4309] | 0.7579 |
| Children's Social Care Expenditure (£, Ms) |  |  | 0.8850 [0.1839, 1.5860] | 0.0183 | 1.0783 [0.3041, 1.8526] | 0.0124 |
| Num.Obs. | 1342 | 1342 | 932 | 932 | 932 | 932 |
| R2 | 0.000 | 0.000 | 0.479 | 0.479 | 0.967 | 0.967 |
| R2 Adj. | -0.139 | -0.139 | 0.366 | 0.366 | 0.960 | 0.960 |
| AIC |  |  |  |  | 8222.5 | 8222.5 |
| BIC |  |  |  |  | 9035.1 | 9035.1 |
| Log.Lik. |  |  |  |  | -3943.229 | -3943.229 |
| F |  |  |  |  | 135.600 | 135.600 |
| LA Fixed Effects | Yes | Yes | Yes | Yes | Yes | Yes |
| Time Fixed Effects | Yes | Yes | Yes | Yes | Yes | Yes |
| Clustered Standard Errors | Yes | Yes | Yes | Yes | Yes | Yes |
| Table reports results from multivariate longitudinal regression models. | | | | | | |
| Robust SEs are clustered at LA level and use a bias-reduced linearization estimator (CR2) | | | | | | |

## Analysis on subgroup of children who have been in care for at least 2.5 years

We supplement our results with an analysis of the stability of placements for the cohort of children who have been in care for at least 2.5 years.

First, we compare the relationship between the values and annual changes for the stability measure used in the manuscript (% of long-term placements relative to all children in care) and the long-term stability of children in care who have been looked after for at least 2.5 years (available only from 2018-22). The figures show a clear association between the two.

### Figure A8


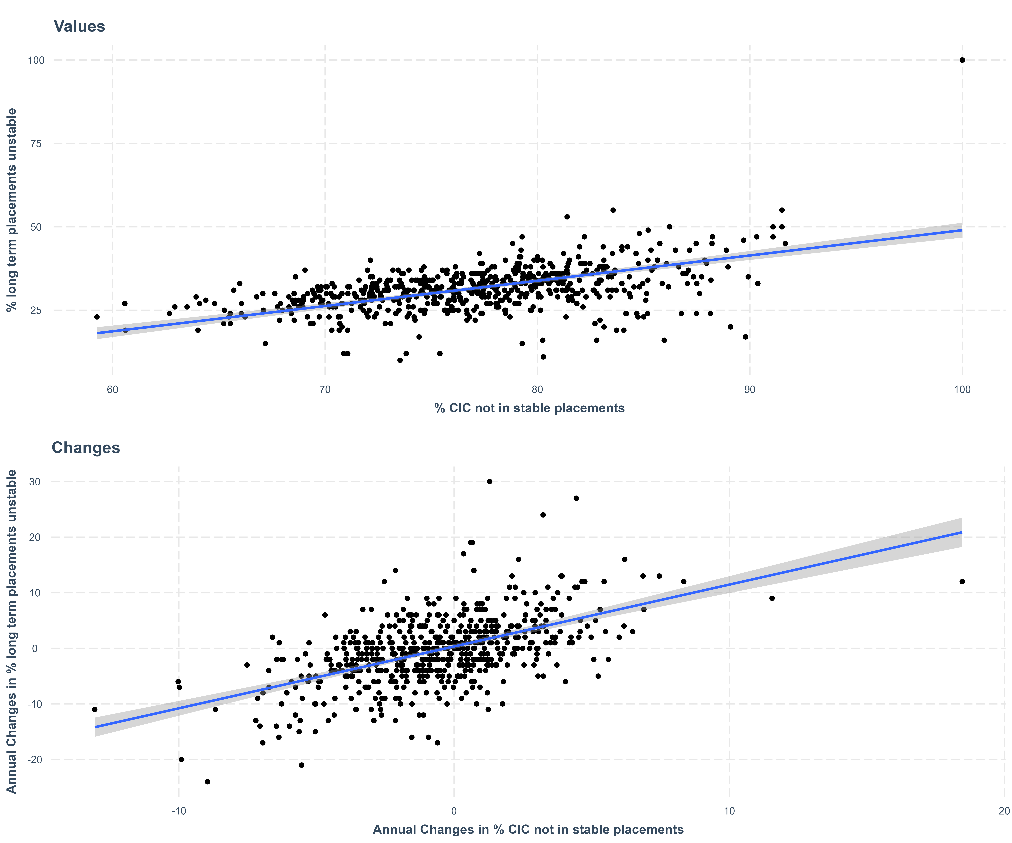


Next, we reproduce figure 2 in the paper, showing the association between average changes in for-profit outsourcing and placement stability between 2018 and 2022. We see a similar relationship as LAs with higher increases in for-profit outsourcing have, on average, a higher increase in the percent of children placed in unstable placements.

### Figure A9


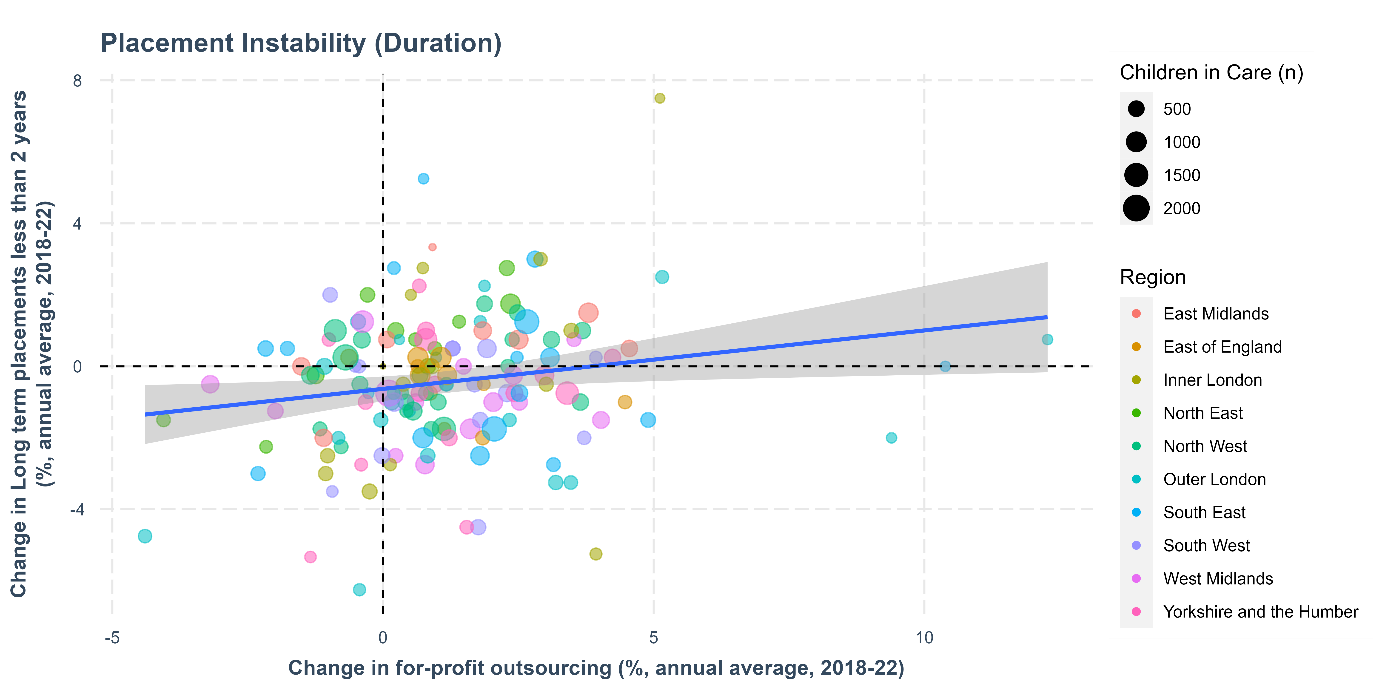


Finally, we reproduce our regression results for this alternative stability measure. We see the same bivariate result – that increases in for-profit outsourcing are associated with decreases, on average, of levels of stability. When we control for covariates in this regression, we observe similar effect sizes but lose statistical significance. Given the shorter period this data is available for (2018-2022 compared to 2011-2022), we have a highly reduced n when including control variables which might partially explain the loss of statistical significance.

### Table A12

|  | **TWFE** | **TWFE** | **TWFE** | **TWFE** | **CBPS** | **CBPS** |
| --- | --- | --- | --- | --- | --- | --- |
|  | **Children in placements for over 2 years (%) [.95 ci]** | **p-value** | **Children in placements for over 2 years (%) [.95 ci]** | **p-value** | **Children in placements for over 2 years (%) [.95 ci]** | **p-value** |
| For-profit Outsourcing (%) | 0.1474 [0.0253, 0.2696] | 0.0338 | 0.1045 [-0.0853, 0.2942] | 0.3083 | 0.1180 [-0.0069, 0.2428] | 0.1672 |
| Fostering placements (%) |  |  | -0.1967 [-0.4541, 0.0606] | 0.1390 | -0.2032 [-0.4508, 0.0443] | 0.1153 |
| CIC ethnicity (white, %) |  |  | 0.0971 [-0.2280, 0.4222] | 0.5608 | 0.0138 [-0.3307, 0.3584] | 0.9378 |
| CIC gender (Female, %) |  |  | -0.1857 [-0.5713, 0.1999] | 0.3492 | -0.2162 [-0.6007, 0.1682] | 0.2773 |
| CIC (n) |  |  | 0.0018 [-0.0100, 0.0137] | 0.7659 | 0.0011 [-0.0099, 0.0120] | 0.8533 |
| Children's Social Care Expenditure (£, Ms) |  |  | 0.0705 [-0.0827, 0.2237] | 0.3752 | 0.0770 [-0.0881, 0.2421] | 0.3969 |
| Num.Obs. | 747 | 747 | 594 | 594 | 594 | 594 |
| R2 | 0.019 | 0.019 | 0.039 | 0.039 | 0.595 | 0.595 |
| R2 Adj. | -0.243 | -0.243 | -0.312 | -0.312 | 0.446 | 0.446 |
| AIC |  |  |  |  | 3760.6 | 3760.6 |
| BIC |  |  |  |  | 4466.9 | 4466.9 |
| Log.Lik. |  |  |  |  | -1719.321 | -1719.321 |
| F |  |  |  |  | 4.007 | 4.007 |
| LA Fixed Effects | Yes | Yes | Yes | Yes | Yes | Yes |
| Time Fixed Effects | Yes | Yes | Yes | Yes | Yes | Yes |
| Clustered Standard Errors | Yes | Yes | Yes | Yes | Yes | Yes |
| Table reports results from multivariate longitudinal regression models. | | | | | | |
| Robust SEs are clustered at LA level and use a bias-reduced linearization estimator (CR2) | | | | | | |
